# Interaction of c-Cbl with Myosin IIA Regulates Bleb Associated Macropinocytosis of Kaposi's Sarcoma-Associated Herpesvirus

**DOI:** 10.1371/journal.ppat.1001238

**Published:** 2010-12-23

**Authors:** Mohanan Valiya Veettil, Sathish Sadagopan, Nagaraj Kerur, Sayan Chakraborty, Bala Chandran

**Affiliations:** H. M. Bligh Cancer Research Laboratories, Department of Microbiology and Immunology, Chicago Medical School, Rosalind Franklin University of Medicine and Science, North Chicago, Illinois, United States of America; Oregon Health & Science University, United States of America

## Abstract

KSHV is etiologically associated with Kaposi's sarcoma (KS), an angioproliferative endothelial cell malignancy. Macropinocytosis is the predominant mode of in vitro entry of KSHV into its natural target cells, human dermal microvascular endothelial (HMVEC-d) cells. Although macropinocytosis is known to be a major route of entry for many viruses, the molecule(s) involved in the recruitment and integration of signaling early during macropinosome formation is less well studied. Here we demonstrate that tyrosine phosphorylation of the adaptor protein c-Cbl is required for KSHV induced membrane blebbing and macropinocytosis. KSHV induced the tyrosine phosphorylation of c-Cbl as early as 1 min post-infection and was recruited to the sites of bleb formation. Infection also led to an increase in the interaction of c-Cbl with PI3-K p85 in a time dependent manner. c-Cbl shRNA decreased the formation of KSHV induced membrane blebs and macropinocytosis as well as virus entry. Immunoprecipitation of c-Cbl followed by mass spectrometry identified the interaction of c-Cbl with a novel molecular partner, non-muscle myosin heavy chain IIA (myosin IIA), in bleb associated macropinocytosis. Phosphorylated c-Cbl colocalized with phospho-myosin light chain II in the interior of blebs of infected cells and this interaction was abolished by c-Cbl shRNA. Studies with the myosin II inhibitor blebbistatin demonstrated that myosin IIA is a biologically significant component of the c-Cbl signaling pathway and c-Cbl plays a new role in the recruitment of myosin IIA to the blebs during KSHV infection. Myosin II associates with actin in KSHV induced blebs and the absence of actin and myosin ubiquitination in c-Cbl ShRNA cells suggested that c-Cbl is also responsible for the ubiquitination of these proteins in the infected cells. This is the first study demonstrating the role of c-Cbl in viral entry as well as macropinocytosis, and provides the evidence that a signaling complex containing c-Cbl and myosin IIA plays a crucial role in blebbing and macropinocytosis during viral infection and suggests that targeting c-Cbl could lead to a block in KSHV infection.

## Author Summary

KSHV is etiologically associated with Kaposi's sarcoma (KS), the most common AIDS related neoplasm. The first key step in KSHV infection is its initial contact with target cells and entry. While it is known that KSHV uses macropinocytosis for its infectious entry into its natural target cells, HMVEC-d cells, we know little about the molecule(s) involved in this event. Here, we show that the adaptor protein c-Cbl plays a major role in regulating bleb associated macropinocytosis of KSHV. The results demonstrate that c-Cbl protein functions as an adaptor for the myosin II hexameric complex in macropinocytic events. Knocking down c-Cbl by shRNA induces defects in myosin II dependent blebbing and KSHV entry, indicating that c-Cbl uses myosin II to coordinate signaling pathways, resulting in bleb formation and bleb retraction. This work provides a clear understanding of the role of c-Cbl in the recruitment and integration of signaling molecules around the macropinosome during virus infection, and identifies potential targets to intervene in KSHV infection.

## Introduction

KSHV is etiologically associated with Kaposi's sarcoma (KS), the most common AIDS related malignancy, as well as with two lymphoproliferative diseases, primary effusion lymphoma (PEL) and multicentric Castleman's disease [1], [2]. KSHV infects a variety of target cells both *in vivo* and *in vitro*. Entry into the target cells is the most crucial step in the establishment of a successful infection for all viruses. KSHV utilizes different modes of endocytosis to enter different target cells *in vitro* [3]. For example, KSHV enters human foreskin fibroblasts (HFF) via clathrin mediated endocytosis and enters HMVEC-d cells via macropinocytosis [3], [4], [5]. During the early stages of infection of HMVEC-d cells, KSHV forms a multi-molecular complex with host cell heparan sulfate, integrins (α3β1, αVβ3 and αVβ5) and transporter protein xCT with the subsequent induction of overlapping signal cascades [3]. Our studies show that KSHV induces a complex set of signaling molecules that are involved in diverse biological functions to regulate the various aspects of KSHV endocytosis including internalization, trafficking in the cytoplasm and nuclear delivery [3]. KSHV activates FAK, Src, PI3-K, Rho-GTPases and cytoskeleton rearrangement which are all critical for entry of virus [6], [7], [8], [9]. KSHV also activates other downstream molecules such as PKC-ζ, MEK, ERK1/2 and NFkB which are essential for viral gene expression [6], [7], [8], [9].

The Cbl family of adaptor proteins include three mammalian isoforms, c-Cbl, Cbl-b and Cbl-c or Cbl-3 [10], [11]. Cbl proteins play important roles in signal transduction as negative regulators by mediating the ubiquitinilation and down-regulation of proteins while it acts as a positive regulator through their scaffold function in assembling signaling complexes [10], [11]. c-Cbl has been shown to bind to several molecules critical in signal transduction [10], [11]. Tyrosine phosphorylation of c-Cbl has been shown to be crucial for c-Cbl mediated adaptor functions in most circumstances [11], [12], [13]. However, the adaptor functions of c-Cbl and a c-Cbl mediated signaling pathway during virus infection has not been demonstrated.

Macropinocytosis provides a major route for the productive infection of many viruses including KSHV. Macropinocytosis is an actin dependent membrane associated process which involves recruitment and integration of several signaling molecules necessary for cytoskeletal rearrangement and membrane remodeling. However, there is little information about the molecules involved in the recruitment and integration of signaling during macropinosome formation. Even though c-Cbl has been shown to recruit and link different signaling molecules in a signaling pathway, a direct role for c-Cbl in the process of macropinocytosis has not been established yet.

Here we identified that c-Cbl is involved in KSHV entry and critical for triggering the macropinocytic event. Our data provide evidence that the interaction between c-Cbl and myosin IIA, a motor protein that binds to the proline rich domain of c-Cbl, regulates macropinocytosis of KSHV. This study on the functional organization of the c-Cbl and myosin IIA complex and its effect on viral entry provide an important insight into understanding the role of c-Cbl in virus infection.

## Materials and Methods

### Cells and virus

HMVEC-d cells (CC-2543; Clonetics, Walkersville, Md) were grown in endothelial cell medium (EBM2; Cambrex, Walkersville, MD). Induction of the KSHV lytic cycle in BCBL-1 cells, supernatant collection, and virus purification procedures were described previously [14]. KSHV DNA was extracted from the virus, and the copies were quantitated by real-time DNA PCR using primers amplifying the KSHV ORF 73 gene as described previously [15].

### Generation of HMVEC-d cells expressing shRNA

A pool of lentivirus shRNA specific for human c-Cbl and non-specific control shRNA were purchased from Santa Cruz Biotechnology (Santa Cruz, CA). HMVEC-d cells were transduced with control lentivirus shRNA and c-Cbl lentivirus shRNA according to the manufacturer's instructions and selected by puromycin hydrochloride (10 µg ml^−1^; Santa Cruz Biotechnology).

### Antibodies and reagents

The following antibodies were used: mouse anti-c-Cbl, mouse anti-phospho Cbl 700 (phosphorylated at Tyr700), and mouse anti-p85 (PI-3K) antibodies (BD Transduction Laboratories, San Diego, CA); anti-phospho MLC II, anti-phospho Cbl 731, anti-phospho Cbl 774 (phosphorylated at Tyr731 and Tyr774), isoform specific anti-myosin II heavy chain antibodies myosin IIA, IIB and IIC (Cell Signaling Technology, Danvers, MA); mouse anti-phospho tyrosine (4G10 clone; Millipore, Temecula, CA); mouse anti-tubulin, mouse anti-beta actin antibodies (Sigma, St Louis, MO); rabbit anti-lamin B (Abcam, Cambridge, MA); rabbit anti-HA (Zymed, Invitrogen, Carlsbad, CA); mouse anti-ubiquitin (P4D1), mouse ant-GFP, mouse anti-GST (Santa Cruz, CA); rabbit anti-gB and mouse anti-gpK8.1A antibodies were created in our laboratory[16], [17]; anti-goat, anti-rabbit and anti-mouse antibodies linked to horseradish peroxidase (KPL Inc., Gaithersburg, Md.); DAPI, rhodamine conjugated dextran, Alexa 594 or Alexa 488 conjugated phalloidin and anti-rabbit and anti-mouse secondary antibodies conjugated to Alexa 488, Alexa 594 (Invitrogen); protein A and G–Sepharose CL-4B beads (Amersham Pharmacia Biotech, Piscataway, NJ); blebbistatin, U0126 (Calbiochem, La Jolla, CA); TPA, LY294002 (Sigma).

### Measurement of KSHV entry by real-time DNA PCR

Unless stated otherwise, cells were infected with KSHV at 10 DNA copies (multiplicity of infection [MOI]) per cell at 37°C. Entry was measured by infecting the cells with KSHV for 30 min. The cells were washed with HBSS to remove the unbound virus, treated with 0.25% trypsin-EDTA for 5 min at 37°C to remove the bound but non-internalized virus, and washed. Cells were recovered by centrifugation and total DNA was isolated from infected or uninfected cells using a DNeasy kit (QIAGEN, Valencia, CA) as described previously [15]. To calculate percent of inhibition of KSHV entry, internalized KSHV DNA was quantitated by amplification of the ORF73 gene by real-time DNA PCR [15]. The KSHV ORF73 gene cloned in the pGEM-T vector (Promega) was used for the external standard. The cycle threshold (Ct) values were used to generate the standard curve and to calculate the relative copy numbers of viral DNA in the samples. Percentage inhibition was calculated by considering the ORF73 copy numbers in untransduced cells as 100%.

### KSHV gene expression measurement by real-time RT-PCR

Total RNA was prepared from infected or uninfected cells using an RNeasy kit (QIAGEN) as described previously [15]. To quantitate viral gene expression, isolated RNA was subjected to ORF73 and ORF50 RNA expression by real-time reverse transcription (RT)-PCR using gene specific real-time primers and specific TaqMan probes [15]. The relative copy numbers of the transcripts were calculated from the standard curve plotted using the Ct values for different dilutions of in vitro-transcribed transcripts. These values were normalized to each other using the values of the GAPDH control reactions. Percentage inhibition was calculated by considering ORF73 and ORF50 gene expression in untransduced cells as 100%.

### Western blotting

Cells were lysed in RIPA buffer (15 mM NaCl, 1 mM MgCl2, 1 mM MnCl2, 2 mM CaCl2, 2 mM phenylmethylsulfonyl fluoride, and protease inhibitor mixture (Sigma)) and centrifuged at 12,000 rpm at 4°C for 15 min. Lysates were normalized to equal amounts of protein and the proteins were separated by 7.5–12.5% gradient SDS-PAGE, transferred to nitrocellulose and probed with the indicated primary antibodies. Detection was by incubation with species-specific HRP-conjugated secondary antibodies. Immunoreactive bands were visualized by enhanced chemiluminescence (Pierce, Rockford, IL) according to the manufacturer's instructions. The bands were scanned and quantitated using the FluorChem FC2 and Alpha-Imager Systems (Alpha Innotech Corporation, San Leonardo, CA).

### Immunoprecipitation

Two hundred micrograms of cell lysates prepared as described in the above section were incubated for 2 h with immunoprecipitating antibody at 4°C, and the immune complexes were captured by protein A or G-Sepharose. The samples were tested by Western blot with specific primary and secondary antibodies.

### Mass spectrometry and protein analysis

HMVEC-d cells were infected with KSHV for different time points. The samples were resolved on an SDS-PAGE gel and the gel was stained with Coomassie blue. The bands of interest were excised, digested with trypsin, separated by reverse phase nano-chromatography and analyzed by mass spectrometry.

### Immunofluorescence and microscopy

Immunofluorescence assay was performed using HMVEC-d cells seeded on 8 well chamber slides (Nalge Nunc International). Infected and uninfected cells were fixed with 3% paraformaldehyde for 15 min, permeabilized with 0.2% Triton X-100, and blocked with Image-iTFX signal enhancer (Invitrogen). The cells were then immunostained with primary antibodies against the specific proteins, followed by fluorescent dye-conjugated secondary antibodies. For colocalization with dextran and transferrin, cells were incubated with the fluid-phase marker dextran Texas Red (40 kD, 0.5 mg ml^−1^; Invitrogen) or Alexa 594 transferrin (35 µg ml^−1^; Invitrogen) at 37°C in the presence or absence of KSHV followed by immunostaining with the appropriate antibodies. Cells were imaged with a Nikon fluorescence microscope equipped with a Metamorph digital imaging system. DIC (Differential Interference Contrast) images were acquired with objectives equipped with DIC optics. For confocal analysis, the Olympus Fluoview 300 fluorescence confocal microscope was used for imaging, and analysis was performed using Fluoview software (Olympus, Melville, NY). All experiments were performed at least three times.

### Quantitative analysis of dextran uptake

HMVEC-d cells, incubated with dextran Texas Red (0.5 mg ml^−1^, 40 kD; Invitrogen) and KSHV for 30 min, were washed twice in HBSS. To remove surface bound dextran, cells were treated with 0.25% trypsin-EDTA and the cells were harvested. Quantitative analysis of dextran uptake was determined by counting the number of cells stained positive for dextran under immunofluorescence microscope. At least 10 different microscopic fields of 50 cells each were counted for each experiment and the results displayed as percentage of dextran positive cells.

### Flow cytometry analysis

Flow cytometry analysis was used to quantify the uptake of dextran during KSHV internalization in control shRNA and c-Cbl shRNA transduced cells. Cells were incubated with 500 µg ml^−1^ FITC-dextran in the presence or absence of virus at 37°C for 30 min. The cells were washed, harvested using trypsin EDTA, fixed and analyzed by flow cytometry. Mean fluorescence intensity was determined using a Becton Dickinson FACS system and CellQuest software. Cells incubated with dextran alone were used as controls.

### Plasmids and transfection

HeLa cells (ATCC CCL-2) were cultured in DMEM containing 10% fetal bovine serum. Wild type, mutants and deletion constructs of c-Cbl, c-Cbl C-terminal domain encompassing PRD (Cbl-C) and c-Cbl N-terminal domain (Cbl-N) constructs were generously provided by Dr. Hamid Band [18] (Eppley Institute for Cancer and Allied Diseases, University of Nebraska Medical Center). Cells were transiently transfected with wild type, mutants and deletion constructs. Transfection was performed using 5 µg of plasmid DNA, lipofectamine 2000 (Invitrogen), and Opti-MEM medium (Invitrogen) according to the manufacturer's instructions. After transfection, cells were cultured for 48 h. Cells were then serum starved for 4 h and stimulated with TPA (100 ng ml^−1^) at 37°C for 5 min. Lysis was performed in RIPA buffer plus protease inhibitors. The cell lysate was used for immunoprecipitation and immunoblotting.

### GST pull-down assay

E. coli BL21 (DE3) cells were transformed with pGEX4T.1 GST-Cbl C (Cbl residues 358–906) plasmids which encode regions encompassing the C-terminal PRD domain of c-Cbl and pGEX4T.1 GST-Cbl N which encodes the N-terminal region of c-Cbl. Expression of the GST-Cbl fusion proteins was induced with IPTG (isopropyl-D-1-thiogalactopyranoside) 1 mM for 3 h at 37°C. The bacterial lysates (500 µg) were incubated with glutathione-sepharose beads (GE Healthcare, U.K.) for 2 h at 4°C. The beads were washed with lysis buffer three times. 293T cells were transiently transfected with 2 µg pEGFP C3 myosin IIA plasmids (Addgene). After 48 h of transfection, cells were lysed in RIPA buffer and 500 µg of the lysates were incubated with the glutathione-Sepharose beads bound with the GST-Cbl fusion proteins. The beads and the bound proteins were collected by centrifugation, washed and the interaction of GST-Cbl with myosin IIA was analyzed by SDS-PAGE and Western blotting using anti-GFP antibody.

### Determination of blebbing

HMVEC-d cells infected with KSHV for 5 min were fixed and stained with DAPI. DIC images were acquired and the cells presenting blebs or no blebs were counted visually. At least 10 random microscopic fields per experiment were counted and expressed as a proportion of the total number of DAPI stained cells.

### Cell membrane preparation

Infected and uninfected cells were washed three times with HBSS and lysed in homogenization buffer (250 mM sucrose, 20 mM HEPES, 10 mM KCL, 1 mM EDTA, 1 mM EGTA and protease inhibitors). The homogenate was subjected to centrifugation at 3,000 rpm for 5 min. Post-nuclear supernatant was centrifuged at 8,000 rpm for 5 min at 4°C. The supernatant was again centrifuged at 40,000 rpm for 1 h at 4°C, and the supernatant and the pellet were considered the cytosolic and the membrane fractions, respectively. The membrane pellet was solubilized using RIPA buffer and used for Western blot.

## Results

### KSHV infection induces tyrosine phosphorylation of c-Cbl and association of c-Cbl with PI3-K

**Figure 1:**
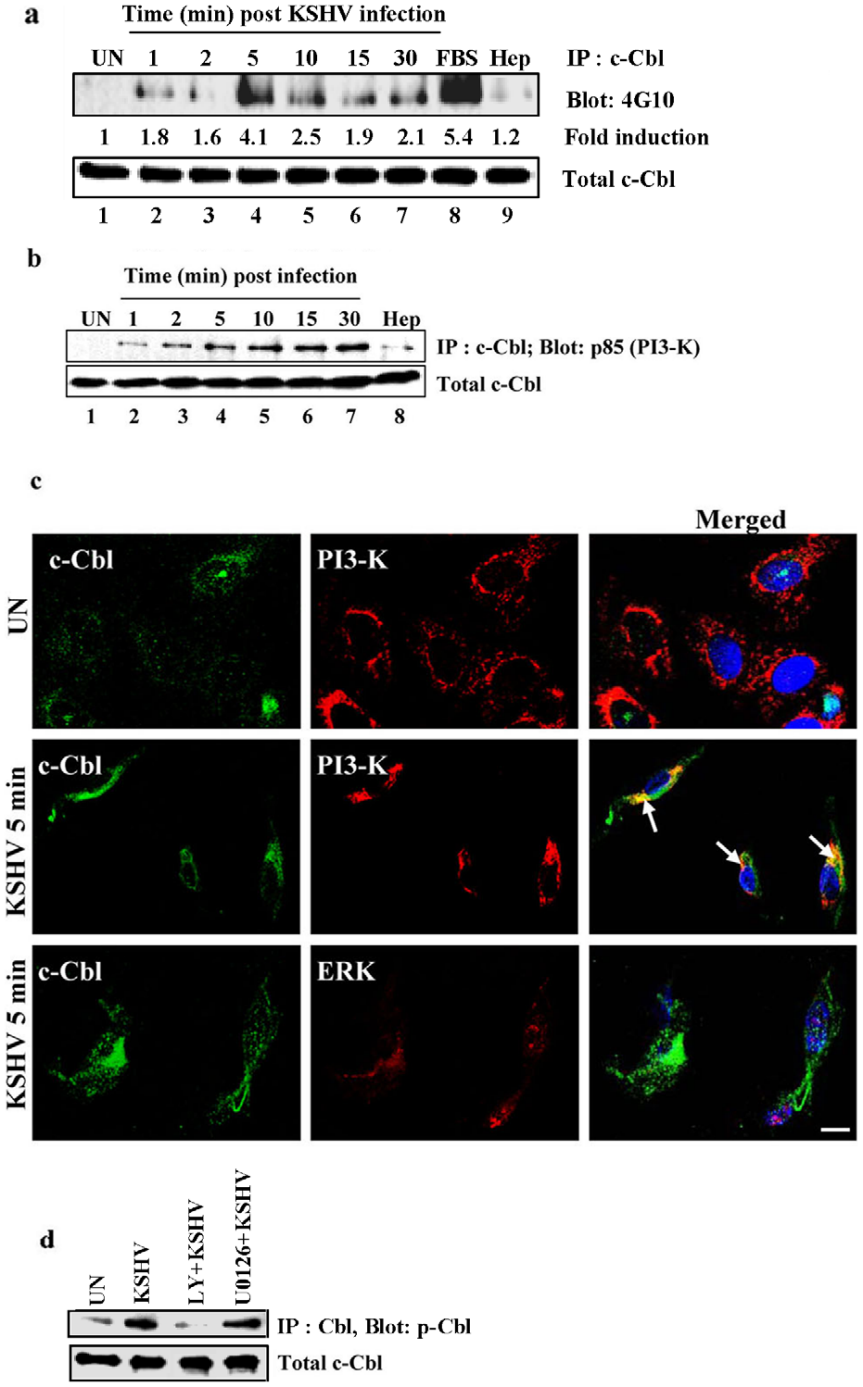
KSHV infection induces tyrosine phosphorylation of c-Cbl and association of c-Cbl with PI3-K. (a) KSHV infection induces tyrosine phosphorylation of c-Cbl. HMVEC-d cells were left uninfected or infected with KSHV for the indicated time points after serum starvation. Tyrosine phosphorylation of c-Cbl was determined by immunoprecipitating with anti-c-Cbl and Western blotting with anti-phosphotyrosine antibody 4G10. Cells treated with 10% FBS for 10 min were used as positive control. As a negative control, cells were infected for 10 min with KSHV pre-incubated with heparin (50 µg ml^−1^) for 1 h at 37°C. Band intensities (middle panel) were assessed as described in the methods and expressed as increased fold tyrosine phosphorylation of c-Cbl over uninfected cells. Bottom panel shows the blot reprobed with anti-c-Cbl antibody. (b) c-Cbl associates with PI3-K in the infected cells. Uninfected or cells infected with KSHV for different time points were lysed and immunoprecipitated with anti-c-Cbl and Western blotted with anti-p85 (PI-3K) antibody. Cells infected for 10 min with heparin treated virus were used as specificity control. Bottom panel shows the blot reprobed with anti-c-Cbl antibody. (c) c-Cbl colocalizes with PI3-K in the infected cells. HMVECd cells infected with KSHV for 5 min were immunostained with anti-c-Cbl and anti-PI3-K p85 antibody and analyzed by confocal microscopy. Arrows indicate colocalization of c-Cbl-with PI3-K p85. ERK staining showing no colocalization with c-Cbl was used as specificity control. (d) Effect of PI3-K and ERK1/2 inhibitors on c-Cbl phosphorylation. HMVEC-d cells either left untreated or pre-treated with 50 µM LY294002 (LY) or 10 µM U0126 for 1 h were infected with KSHV for 10 min. Uninfected and infected cell lysates were immunoprecipitated with anti-c-Cbl and Western blotted with anti-phospho Cbl antibody.

To determine whether c-Cbl and the c-Cbl mediated signaling pathway play roles in KSHV infection, we first examined the early tyrosine phosphorylation kinetics of c-Cbl in KSHV infected cells. HMVEC-d cells infected with KSHV induced rapid tyrosine phosphorylation of c-Cbl, which was detectable as early as 1 min post-infection (p.i.), reaching maximum levels at 5 min (4.1-fold), followed by a decrease which was constent for as much as 30 min p.i. (Figure 1a). To determine whether the phosphorylation of c-Cbl is specifically induced by KSHV, cells were infected with KSHV pre-incubated with heparin. Heparin is known to block the binding of KSHV to the target cells [19]. Compared to the untreated virus, heparin treated virus considerably reduced the phosphorylation of c-Cbl (Figure 1a) which demonstrated the specificity of KSHV induced c-Cbl phosphorylation. The efficient tyrosine phosphorylation of c-Cbl is suggestive of the possible involvement of a c-Cbl mediated signaling pathway in KSHV infection.

We next investigated the link between c-Cbl phosphorylation and other signaling molecules activated during KSHV infection. It is well documented that the interaction of KSHV glycoproteins with integrins and other cellular receptors activate FAK and the downstream molecules Src and PI3-K [7], [9], [14], [20], [21]. c-Cbl has been shown to form a complex with PI3-K p85 in the integrin mediated signaling pathway [12]. We therefore examined whether the association of PI3-K with c-Cbl occurred during KSHV infection. KSHV infection led to an increase in the interaction of c-Cbl with PI3-K p85 in a time dependent manner (Figure 1b). To verify that the c-Cbl-PI3-K interaction is specifically induced by virus, cells were infected with heparin treated virus which notably decreased the association of c-Cbl with PI3-K (Figure 1b). The c-Cbl-PI3-K association was further confirmed by confocal analysis (Figure 1c). Consistent with previous studies [13], our results demonstrated that activated c-Cbl leads to the association of c-Cbl with PI3-K.

Previous studies have shown that ERK1/2 is activated during KSHV infection and is a key signaling molecule implicated in viral gene expression [22]. To examine whether an ERK1/2 associated pathway is involved in c-Cbl mediated signaling, we investigated the association of ERK1/2 with c-Cbl in KSHV infected cells. No colocalization was observed between ERK1/2 and c-Cbl (Figure 1c) which suggested that the ERK associated pathway is not involved in c-Cbl mediated signaling in KSHV infected cells. Taken together, our data suggests that a signaling complex which contains c-Cbl and PI3-K but not ERK1/2 is involved in the integrin mediated signaling pathway of KSHV infection.

To further demonstrate the relationship between the interaction of c-Cb1 with PI3-K but not with ERK1/2, we studied the effect of PI3-K and ERK1/2 inhibitors in KSHV induced c-Cbl phosphorylation. HMVEC-d cells pretreated with the PI3-K inhibitor, LY294002, and the ERK1/2 inhibitor, U0126 were infected with KSHV for 10 min and the lysates were analyzed for c-Cbl phosphorylation. KSHV induced c-Cbl phosphorylation was abolished by the PI3-K inhibitor LY294002, whereas the ERK1/2 inhibitor U0126 did not show any inhibition on c-Cbl phosphorylation (Figure 1d). The failure of ERK1/2 inhibitor to abolish c-Cbl phosphorylation confirmed that ERK1/2 is not associated with c-Cbl induction in KSHV infected cells.

### c-Cbl shRNA inhibits KSHV entry and infection

**Figure 2:**
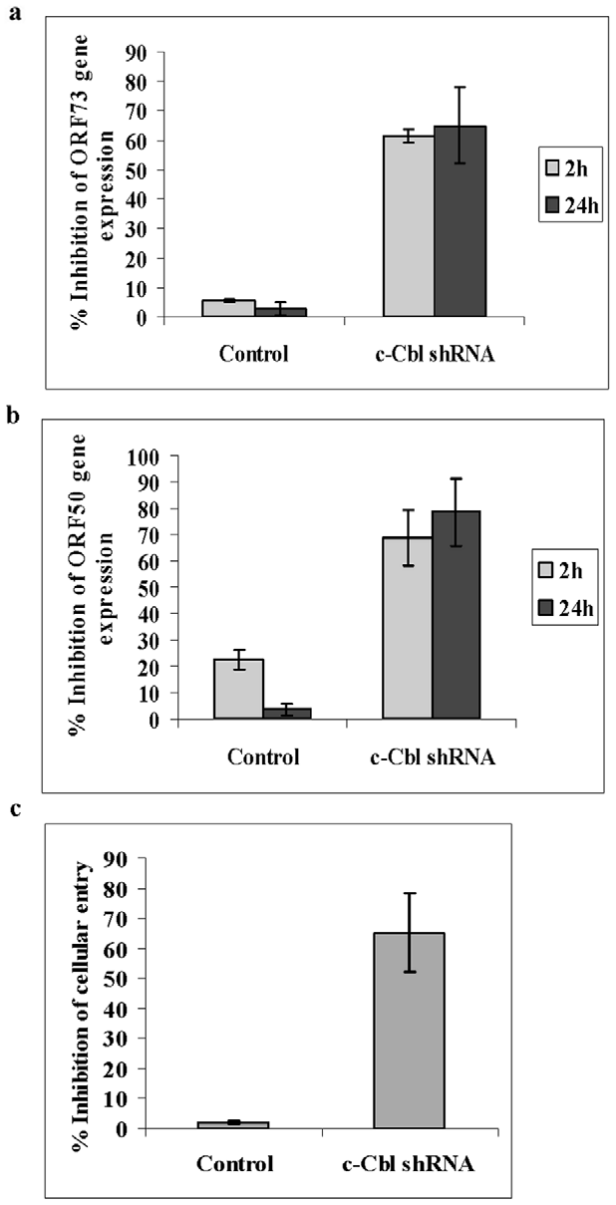
c-Cbl shRNA inhibits KSHV entry and infection. (a and b) KSHV gene expression in c-Cbl ShRNA transduced cells. Untransduced, control shRNA and c-Cbl shRNA transduced HMVEC-d cells were infected with KSHV. At 2 and 24 h post infection, cells were harvested, total RNA isolated and viral gene expression determined by real-time RT-PCR with KSHV ORF73 (a) and ORF50 (b) gene specific primers and TaqMan probes. (c) Entry of KSHV in c-Cbl ShRNA transduced cells. Untransduced, control shRNA and c-Cbl shRNA transduced HMVEC-d cells were infected with KSHV for 30 min. DNA was isolated and KSHV entry was determined by real-time DNA PCR for ORF73 gene. For all the above, each reaction was done in duplicate and each bar represents the average ± SD of three experiments.

We next used c-Cbl lentivirus encoding shRNAs to knockdown c-Cbl activity in HMVEC-d cells (c-Cbl shRNA cells) to analyze the functions of c-Cbl in KSHV infected cells. The c-Cbl specific shRNA inhibited 90% of c-Cbl expression as detected by Western blotting with antibodies to c-Cbl (Figure S1a). Untransduced, control shRNA and c-Cbl shRNA transduced cells were infected with KSHV for 2 and 24 h, and viral gene expression was determined by real-time RT-PCR analysis. Compared with control cells, c-Cbl shRNA transduced cells showed about 60–70% inhibition of the latency associated ORF 73 gene (Figure 2a) and 70–80% inhibition of the lytic switch ORF 50 gene (Figure 2b) expression.

We next determined whether the inhibition of viral-gene expression by c-Cbl shRNA was due to a blockage at the entry stage of the virus. To determine c-Cbl's role in KSHV entry, internalization of viral DNA was determined by measuring viral ORF 73 DNA copy numbers by real-time DNA PCR. We observed ∼65% inhibition of KSHV entry in c-Cbl shRNA cells compared to control cells (Figure 2c). Internalized KSHV ORF 73 DNA copy numbers and ORF 50 and ORF 73 RNA copy numbers are shown as histograms in supplementary Figure S1. Taken together, these studies demonstrated that the decreased viral gene expression observed in c-Cbl shRNA cells was due to a decrease in the entry of KSHV. These results further suggested that a c-Cbl containing signaling complex may be crucial for the initiation of entry and for a productive infection.

### KSHV infection utilizes blebbing as a mechanism of entry in HMVEC-d cells

**Figure 3:**
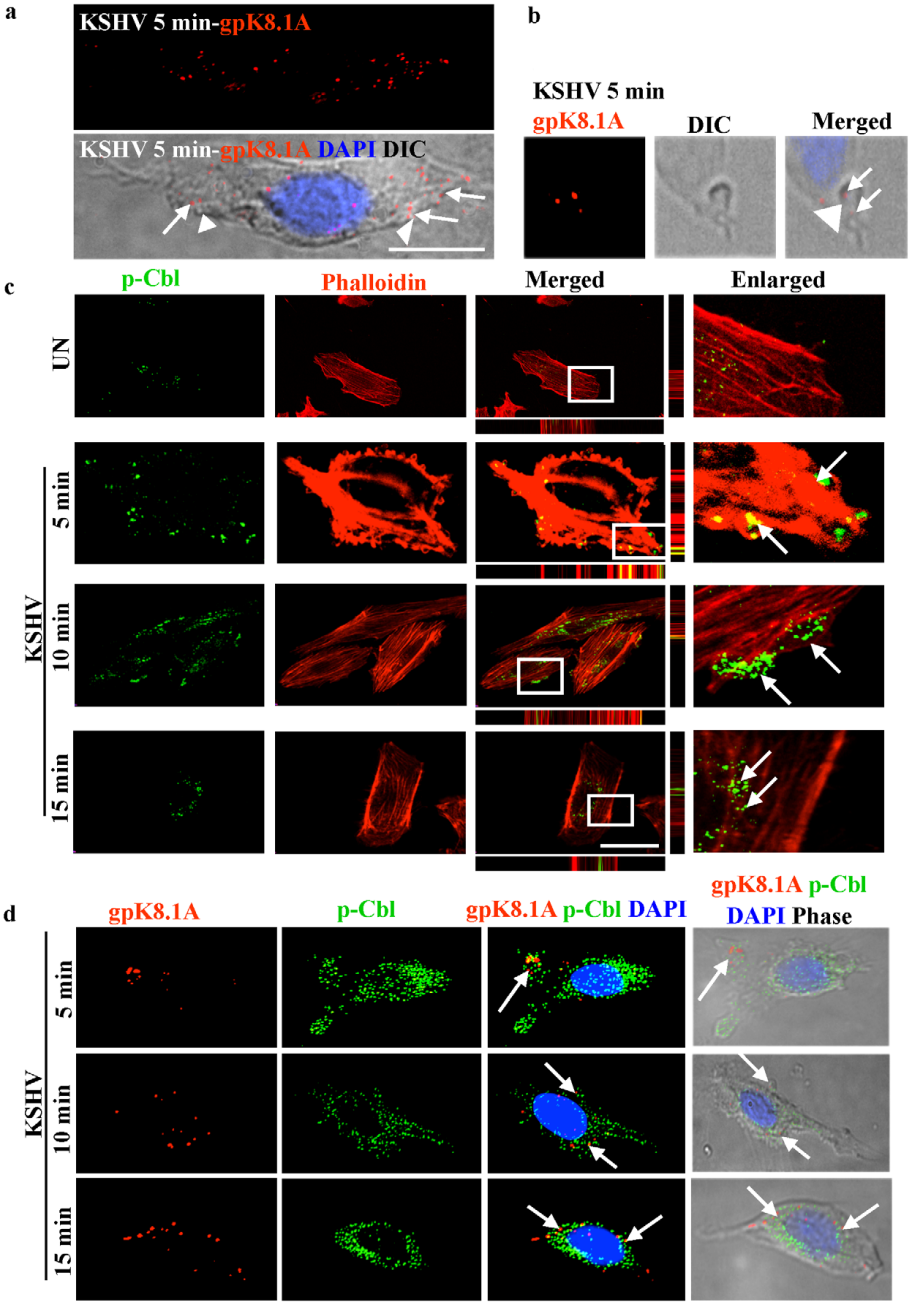
c-Cbl associates with KSHV induced blebs. (a) Association of blebs with KSHV in the infected cells. HMVEC-d cells were infected with KSHV for 5 min and immunostained with anti-gpK8.1A (viral envelope glycoprotein) antibody for the detection of KSHV. Images showing blebs were acquired by fluorescence microscopy equipped with DIC (Differential Interference Contrast). The merged image represents a gpK8.1A, DIC, and DAPI stained nucleus. Arrow heads indicate blebs and arrows indicate viral particles (gpK8.1A staining) associated with the blebs. Scale bar: 10 µm. (b) Image of a single bleb and KSHV (gpK8.1A staining) were acquired by fluorescence microscopy equipped with DIC under an 100x objective lens. The gpK8.1A, DIC and DAPI-stained nucleus are merged in the rightmost image. Arrow heads indicate a bleb and arrows indicate viral particles associated with the bleb. (c) Association of blebs with c-Cbl in the infected cells. Uninfected or cells infected with KSHV for 5, 10 and 15 min were stained with rhodamine-phalloidin and with anti-phospho c-Cbl (p-Cbl) antibody. The staining was analyzed by confocal microscopy sections through the z axis. Arrows indicate the association of p-Cbl with blebs and internalization at different time points. Bottom and side bars represent the xz axis. The boxed areas are enlarged in the rightmost panel. Scale bar: 10 µm. (d) Association of viral particles with c-Cbl in the infected cells. HMVEC-d cells infected with KSHV for 5, 10 and 15 min were fixed and processed for double immunofluorescence using anti-p-Cbl and anti-gpK8.1A antibodies. Panels show the staining of gpK8.1A, and the images merged with p-Cbl, DAPI and DIC. Arrows indicate the colocalization of p-Cbl and gpK8.1A in the infected cells.

In our earlier studies we have demonstrated that macropinocytosis is the major pathway of KSHV entry leading to a productive infection in HMVEC-d cells [4]. Since c-Cbl inhibited KSHV's entry, we theorized that c-Cbl might be playing a role in macropinocytosis and associated signaling events. Viruses such as vaccinia virus that use macropinocytosis as a mode of entry induce signaling molecules and cytoskeletal rearrangements in the form of blebs which ultimately retract and ingest viral particles [23], [24]. To determine whether blebs were involved in KSHV infection, we used DIC image analysis and observed the association of KSHV with blebs. As shown in Figure 3a, bleb formation and the association of individual blebs with KSHV was observed by 5 min p.i.. The DIC microscopic analysis of a single bleb for viral particles confirmed that the blebs, formed during viral infection, were associated with viral particles (Figure 3b). To further investigate whether KSHV infection induces blebbing, HMVEC-d cells were infected with KSHV for 2 and 5 min and then actin, a well known determinant of cell shape and blebbing, was stained with phalloidin [25]. Within 2 min of infection, membrane protrusions appeared along the cell surface which rapidly enlarged into well-formed blebs at 5 min (Figure S2). We also observed the association of viral particles at the bleb forming site as well as with retracting blebs (Figure S2). Unlike the well formed blebs, the retracting blebs were characterized by a thick actin cortex [26]. These results demonstrated that early during infection, KSHV induces actin reorganization and the subsequent formation of blebs that may be involved in its entry.

### c-Cbl associates with KSHV induced membrane blebs

Since we observed that c-Cbl shRNA inhibited KSHV entry, which involves bleb formation, we hypothesized that c-Cbl and its phosphorylation might be involved in the dynamics of virus induced blebbing. To understand the function of c-Cbl in blebbing, we examined the localization of phosphorylated c-Cbl (p-Cbl) in KSHV infected cells. Confocal microscopy analysis showed that KSHV induced p-Cbl localized to blebs as early as 2 min p.i. (data not shown). Bleb formation, and its association with p-Cbl, was maximal at 5 min, and by 10 min blebs containing p-Cbl started internalizing and p-Cbl was mostly observed at the nuclear periphery by 15 min p.i. (Figure 3c). A similar pattern of localization was exhibited by p-Cbl and virus at the blebs as early as 5 min p.i., and accumulation of p-Cbl and virus around the nuclear periphery was observed at 15 min p.i. (Figure 3d). These results suggested that the recruitment of phosphorylated c-Cbl to the sites of bleb formation was involved in bleb associated entry of the virus.

### c-Cbl is essential for macropinocytosis of KSHV

**Figure 4:**
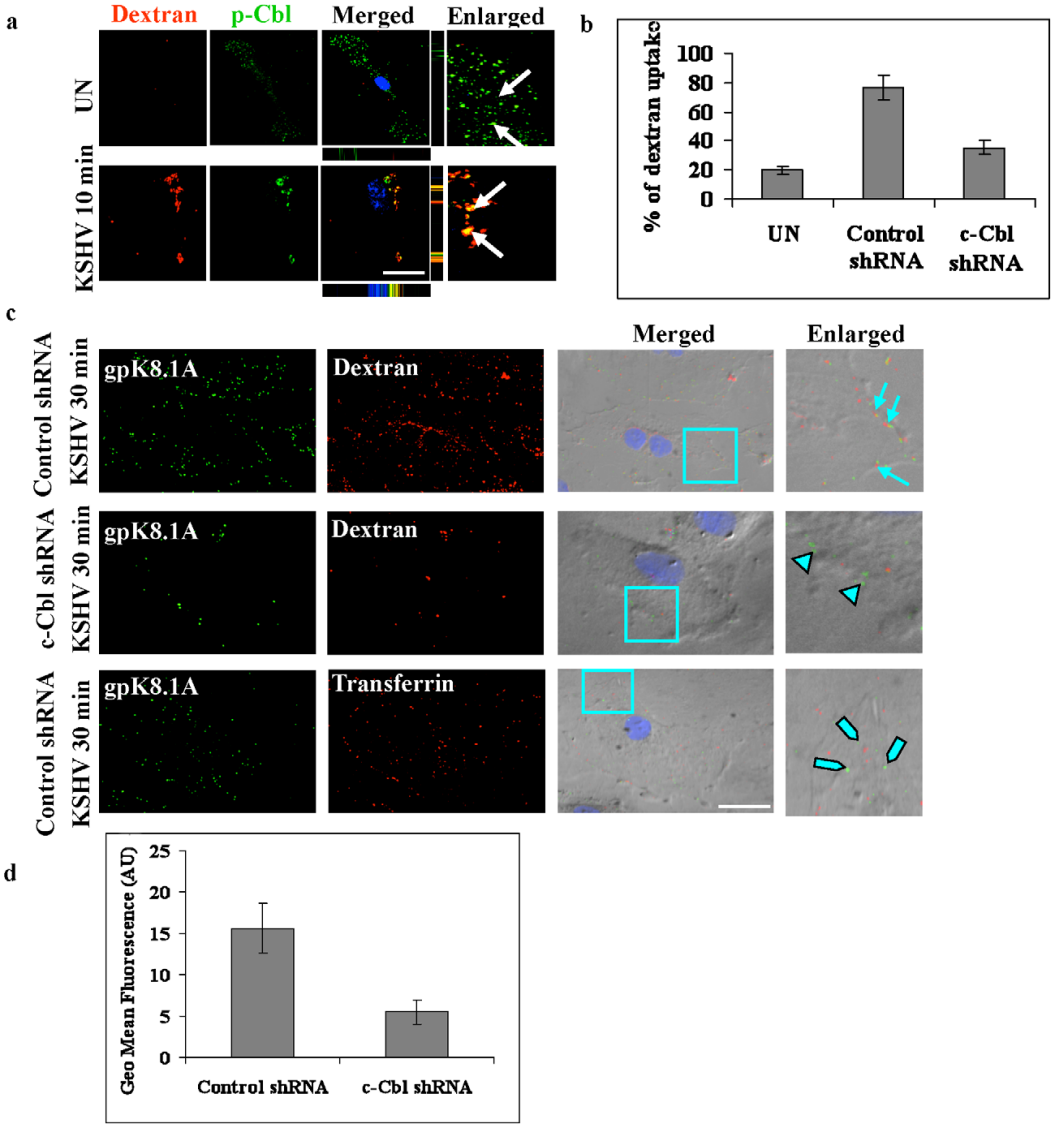
c-Cbl is involved in macropinocytosis of KSHV. (a) Colocalization of c-Cbl with the macropinocytosis marker dextran. HMVEC-d cells were incubated with medium containing Texas Red labeled dextran alone (no virus, UN) or with KSHV and Texas Red labeled dextran at 37°C for 10 min. Cells were processed for confocal immunofluorescence using anti-p-Cbl antibody and costained with DAPI. Arrows in the merged panel indicate colocalization of p-Cbl with dextran. Scale bar: 10 µm. Bottom and side bars represent the xz axis. (b) Dextran uptake in c-Cbl shRNA cells. Control shRNA or c-Cbl shRNA transduced HMVEC-d cells were incubated with KSHV and Texas Red labeled dextran or with Texas Red labeled dextran alone (UN) at 37°C for 30 min. Quantitative analysis of dextran uptake was determined as described in the methods and represent the mean ± SD of three independent experiments. (c) Colocalization of dextran and KSHV. HMVEC-d cells transduced with control shRNA or c-Cbl shRNA were incubated with KSHV and Texas Red labeled dextran for 30 min at 37°C. Control shRNA cells incubated with KSHV and Alexa 594 labeled transferrin for 30 min were used as specificity controls. The cells were then stained with anti-gpK8.1A antibody and analyzed by fluorescence microscopy combined with DIC. Merged panel shows the images merged with DAPI stained nuclei and DIC. The boxed areas are enlarged in the rightmost panel. Arrows: colocalization of KSHV with dextran in the control shRNA cells. Arrow heads: viral particles remained at the cell membrane in c-Cbl shRNA cells. Blocked arrows: non-colocalization of viral particles with transferrin in control shRNA cells. (d) Flow cytometry analysis of FITC-dextran uptake. Control and c-Cbl shRNA cells were incubated with 500 µg ml^−1^ FITC-dextran in the presence or absence of virus at 37°C for 30 min. The cells were then washed, harvested, fixed and analyzed by flow cytometry. Quantification of flow cytometry analysis of dextran uptake is shown as mean fluorescence intensity.

To further explore the role of c-Cbl in bleb associated macropinocytosis, we performed a confocal immunofluorescence colocalization study between p-Cbl and the macropinocytosis marker dextran in infected cells. This analysis showed that dextran colocalized with p-Cbl at 10 min p.i. (Figure 4a) in the infected cells. Next, we performed a dextran uptake study since the uptake of dextran has been used as a biochemical marker of macropinocytosis. We incubated the cells with dextran in the presence or absence of virus for 30 min and then quantitated the level of uptake. As shown in Figure 4b and supplementary information Figure S3a, c-Cbl shRNA cells showed a drastic inhibition of dextran uptake compared to control shRNA cells infected with KSHV. This indicated that the uptake of dextran or macropinocytosis in KSHV infected cells was a c-Cbl dependent process.

The uptake of dextran and colocalization with KSHV in non-specific control shRNA and c-Cbl shRNA cells were confirmed by immunofluorescence colocalization and DIC analysis. In control shRNA cells infected with KSHV, intracellular KSHV was highly colocalized with dextran, whereas in c-Cbl shRNA cells infected with KSHV, most of the viral particles remained at the membrane periphery although minimal colocalization of KSHV with dextran was observed in some cells (Figure 4c and Figure S3b). Control shRNA cells incubated with KSHV and Alexa 594 transferrin, a marker for clathrin-mediated endocytosis, did not show any significant colocalization with KSHV (Figure 4c and Figure S3b) which demonstrated the specificity of macropinocytosis mediated entry in HMVEC-d cells [4]. These results were consistent with the results of the dextran uptake study, confirming that c-Cbl was critical for inducing the macropinocytic process that promoted the internalization of KSHV.

The uptake of dextran in control shRNA and c-Cbl shRNA cells was further quantified by FACS analysis. Cells were incubated with dextran in the presence or absence of virus for 30 min and the uptake was measured using flow cytometry. As shown in Figure 4d, compared to the control shRNA cells, c-Cbl shRNA cells showed a notable decrease in mean fluorescence intensity. These results are consistent with the results of the immunofluorescence analysis and thus confirmed the role of c-Cbl in KSHV induced macropinocytosis.

### KSHV induces the interaction of c-Cbl with myosin IIA

c-Cbl is a multi-domain protein that interacts with a number of signaling molecules and performs multiple functions [10], [11]. To decipher the molecular partners interacting with c-Cbl during KSHV infection, we used mass spectrometric analysis. HMVEC-d cells were infected with KSHV for 1, 5 and 10 min, lysed and the lysates were immunoprecipitated with anti-c-Cbl antibodies. Samples were separated by SDS-PAGE, followed by Coomassie blue staining and mass spectrometry analysis. Mass spectrometry identified several novel c-Cbl interacting proteins in the infected samples (Supplementary Table S1). The most prominent protein identified in the infected samples was myosin IIA which is one of three isoforms of the non-muscle myosin II family of proteins [27], [28]. The other novel interacting partners of c-Cbl in the infected cells included vimentin, HSP70, BiP protein, Rho GEF and a solute carrier anion exchanger (Table S1).

**Figure 5:**
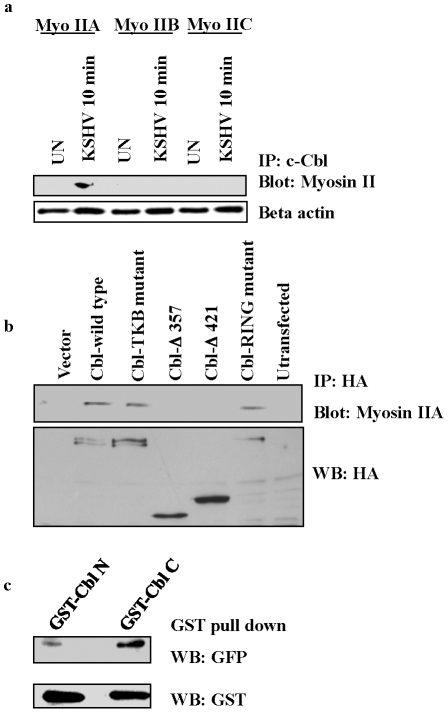
c-Cbl associates with myosin IIA in KSHV infected cells. (a) c-Cbl co-immunoprecipitates with myosin IIA in KSHV infected cells. Serum starved HMVEC-d cells were left uninfected (UN) or infected with KSHV for 10 min. The cells were lysed, immunoprecipitated with anti-c-Cbl antibody and Western blotted with myosin II isoform specific antibodies, IIA, IIB and IIC. Bottom panel: loading control with anti-beta actin antibody. (b) c-Cbl associates with endogenous myosin IIA in HeLa cells. HeLa cells were transfected with HA-tagged empty vector or Cbl wild-type, TKB mutant or RING mutant, truncated Cbl-Δ421 or Cbl-Δ357 constructs. Cells were stimulated with TPA for 5 min and the resulting cell lysates were subjected to immunoprecipitation with anti-HA antibodies followed by immunoblotting with myosin IIA antibody (top). Western blotting was carried out with anti-HA antibody using the same lysates (bottom). (c) GST pull-down assay. GST Cbl-C and Cbl-N fusion proteins expressed in bacterial cells were adsorbed with GST-beads at 4°C for 2 h. The beads were washed and incubated with lysates of 293T cells transiently expressing GFP-tagged myosin IIA. The proteins bound with GST beads were analyzed by Western blotting with anti-GFP antibody. Bottom panel shows GST expression with anti-GST antibody.

To confirm the mass spectrometry data, uninfected and KSHV infected cell lysates were immunoprecipitated with anti-c-Cbl antibody and blotted for the three isoforms of the non-muscle myosin II family, IIA, IIB and IIC, with isoform specific antibodies. Our results confirmed that c-Cbl interacts with myosin IIA in the lysates of infected cells, whereas the other isoforms did not show any interaction with c-Cbl (Figure 5a).

To elucidate the functional domain of c-Cbl involved in myosin IIA interaction, a series of truncated and mutant constructs of c-Cbl with HA epitope tags were used (Figure S4). Since HMVEC-d cells are not easily transfectable, we used HeLa cells for this study. HeLa cells were transfected with vector alone, Cbl wild-type, Cbl-tyrosine kinase binding domain (TKB) mutant, RING domain mutant, and two truncation mutants (Cbl-Δ357 and Cbl-Δ421). As TPA (phorbol ester) has been shown to induce membrane blebbing [29], we used TPA induced HeLa cells to analyze the interaction of over-expressed c-Cbl with endogenous myosin IIA. Transfection of the Cbl-TKB mutant and RING mutant induced the interaction of c-Cbl with myosin IIA similar to full length wild-type Cbl. The truncated versions Cbl-Δ357 and Cbl-Δ421 lacking a C-terminal proline rich domain (PRD) decreased the interaction with myosin IIA considerably (Figure 5b). Expression of all constructs determined by Western blotting with HA revealed comparable levels of protein (Figure 5b). Taken together, these results indicated that the C-terminal region encompassing the PRD of c-Cbl was sufficient for association with myosin IIA.

To further confirm that the C-terminal PRD of c-Cbl interacts with myosin, an in vitro binding assay was performed using bacterially expressed GST fusion proteins of c-Cbl C-terminal (Cbl-C, encompassing PRD) and N-terminal (Cbl-N) domains. GST Cbl-C and Cbl-N proteins adsorbed on glutathione sepharose beads were incubated with 293T cell lysates expressing GFP-tagged myosin IIA. The interaction between GFP-myosin IIA and GST-Cbl was analyzed by Western blotting with anti-GFP antibody. Our results demonstrated that myosin IIA predominantly interacted with Cbl-C domains compared to Cbl-N (Figure 5c).

### Myosin II inhibitor reduces entry of KSHV

**Figure 6:**
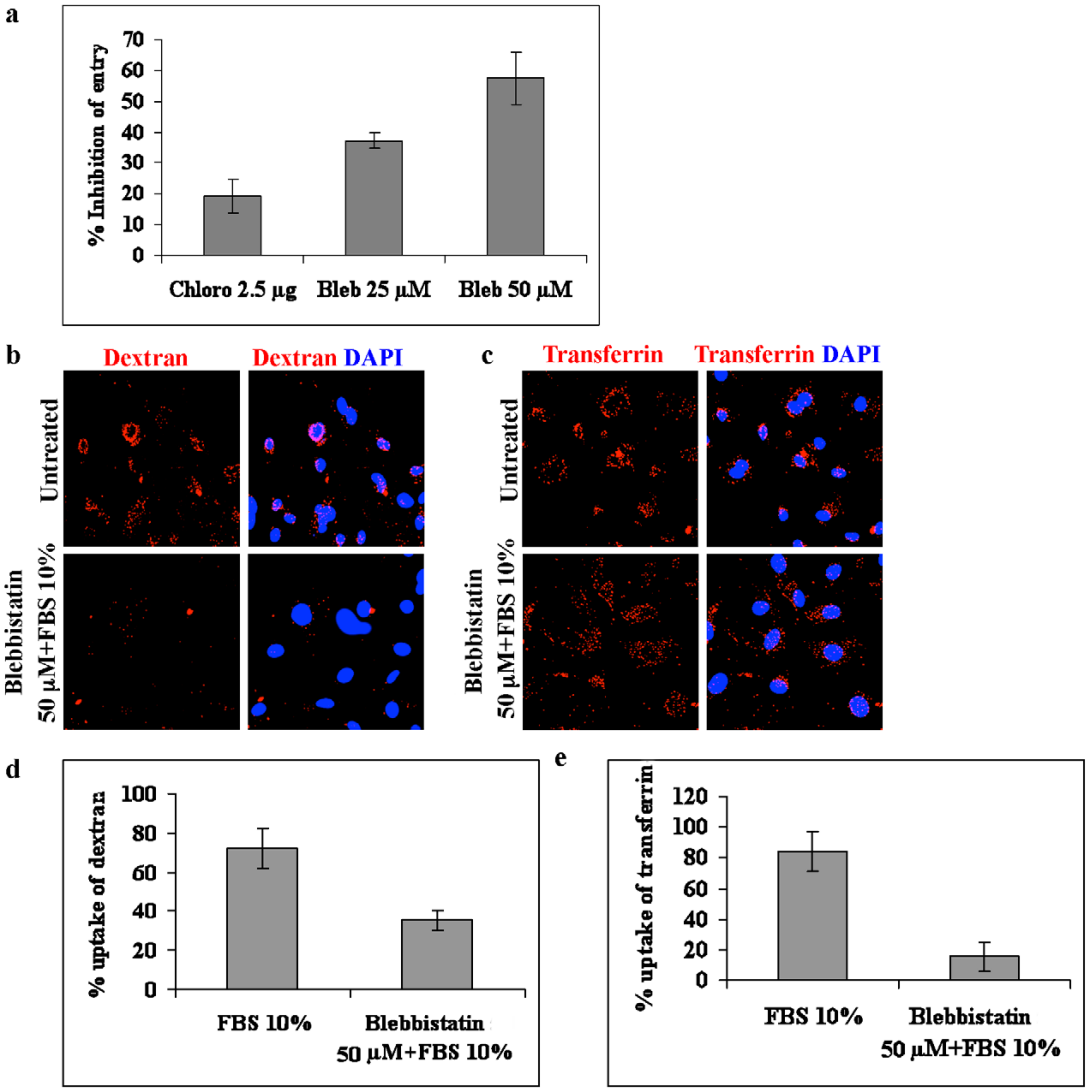
Blebbistatin inhibits macropinocytosis of KSHV. (a) Effect of blebbistatin on entry of KSHV. HMVEC-d cells were pretreated with 25 and 50 µM blebbistatin (Bleb) or with 2.5 µg ml^−1^ chlorpromazine (Chlor) for 1 h before infection with KSHV for 30 min. The cells were then treated with trypsin-EDTA, harvested, and total DNA was isolated. Entry was determined by estimating ORF73 DNA copies by real-time DNA PCR. Percentage inhibition of KSHV DNA internalization was calculated compared to the untreated cells. (b and c). Effect of blebbistatin on dextran and transferrin uptake. HMVEC-d cells were left untreated or treated with blebbistatin for 1 h at 37°C. The cells were then induced with FBS in the presence of dextran (b) or transferrin (c) for 30 min, fixed and analyzed by immunofluorescence. (d and e) Quantitative analysis of dextran and transferrin uptake was determined by counting the number of positive cells. At least 10 different microscopic fields of 50 cells each were counted for each experiment and the results displayed as percentage of dextran or transferrin positive cells.

The interaction of myosin IIA with c-Cbl suggested that their association could be playing a role in blebbing and macropinocytosis of KSHV. To investigate this, we used blebbistatin, a specific inhibitor of myosin II ATPase activity that has been shown to inhibit myosin II induced blebbing [30], [31] and macropinocytosis [23]. As shown in Figure 6a, we observed a dose dependent inhibition of KSHV internalization in 25 µM (∼35%) and 50 µM (∼60%) concentrations of blebbistatin indicating that the entry process was dependent on myosin II activity. As reported previously [4], chlorpromazine, an inhibitor of clathrin dependent endocytosis, did not show any notable decrease in entry of KSHV (Figure 6a). These findings suggested that c-Cbl associated myosin IIA was involved in bleb mediated macropinocytosis of KSHV.

To determine whether blebbistatin treatment affects other internalization pathways, we investigated the effect of blebbistatin on clathrin-mediated internalization, a major and well characterized endocytic pathway of eukaryotic cells. To study this, untreated or blebbistatin treated HMVEC-d cells were induced with FBS in the presence of Alexa 594 labelled transferrin or Texas Red labelled dextran. The endocytic uptake of transferrin and dextran were then analyzed using immunofluorescence. As indicated in Figure 6b and d, blebbistatin strongly inhibited the uptake of dextran, whereas the uptake of transferrin was unaffected (Figure 6c and e). This demonstrated that blebbistatin specifically inhibits macropinocytosis but not clathrin mediated endocytosis pathways.

### c-Cbl is upstream of myosin IIA in the induction of blebs during KSHV infection

**Figure 7:**
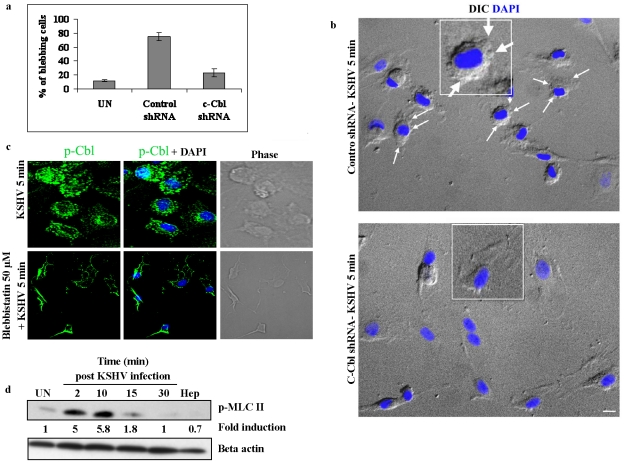
c-Cbl is upstream of myosin IIA in the induction of blebs. (a) Effect of c-Cbl shRNA on KSHV induced blebbing. Control shRNA and c-Cbl shRNA cells were infected with KSHV for 5 min. Uninfected and infected cells were fixed and the blebs were observed under an immunofluorescence microscope with DIC. The percentage of blebbed cells was determined and represented as mean ± SD of three independent experiments. (b) Blebbing in control and c-Cbl shRNA transduced cells. Representative DIC images showing bleb formation in control and c-Cbl shRNA cells infected with KSHV. Nuclei were stained with DAPI and merged with DIC images. KSHV induced blebs can be seen in control cells (arrows), whereas bleb formation in c-Cbl shRNA cells is comparatively less. Scale bar: 10 µm. Insets show a single enlarged cell. (c) Blebbistatin does not affect membrane localization of p-Cbl. HMVEC-d cells were pretreated with blebbistatin for 1 h at 37°C. Untreated and cells treated with blebbistatin were infected with KSHV for 5 min, fixed and immunostained for p-Cbl. (d) KSHV induces phosphorylation of p-MLC II. Serum starved HMVEC-d cells were left uninfected or infected with KSHV for 2, 10, 15 and 30 min. Cells infected with heparin treated (Hep) virus were used as negative control. Cell lysates were Western blotted with anti-p-MLC II antibodies (top panel) or anti-β actin antibodies (bottom panel).

The above studies demonstrated that the c-Cbl interacting partner myosin IIA is a biologically significant component of the c-Cbl signaling pathway. We then explored the role of c-Cbl in myosin II induced blebbing in KSHV infected cells. If c-Cbl is an upstream molecule of myosin IIA, the loss of function of c-Cbl should prevent the formation of myosin II mediated blebs in c-Cbl shRNA cells. To test this hypothesis, control shRNA and c-Cbl shRNA transduced HMVEC-d cells were infected with KSHV and the percentage of cells with blebs was quantitated. As expected, in c-Cbl shRNA transduced cells, the blebs were considerably reduced compared to control shRNA cells (Figure 7a and b). This suggested that c-Cbl and associated myosin IIA molecules were linked to induce membrane blebbing in HMVEC-d cells. Our results indicated that c-Cbl plays an upstream role in the regulation of bleb formation which occurs as a result of myosin II induced cortical contractility [25], [26].

To further demonstrate that c-Cbl is upstream to myosin IIA, we infected blebbistatin treated cells with KSHV and the membrane localization of c-Cbl was observed by immunofluorescence. As shown in Figure 7c, blebbistatin did not inhibit the localization of c-Cbl to the plasma membrane, whereas it prevented the formation of blebs in the infected cells. This suggested that myosin IIA was downstream to c-Cbl and was not involved in the localization of c-Cbl to the plasma membrane.

### KSHV infection induces phosphorylation of myosin light chain II

A subclass of myosins, the class II myosins are hexameric motor proteins composed of two identical heavy chains (MHC), and two pairs of light chains (MLC). It has been well accepted that phosphorylation of the myosin light chain II is a major determinant of force generation and actomyosin dynamics during apoptotic membrane blebbing [32], [33]. Hence, we examined phosphorylation of myosin light chain II (p-MLC II) during KSHV infection. Compared to the uninfected cells, KSHV infection results in rapid and strong phosphorylation of MLC II with maximal phosphorylation at 10 min p.i. (5.8-fold increase) and decreased thereafter (Figure 7d). The specificity of virus induced MLC II phosphorylation was shown using heparin treated virus which did not induce MLC II phosphorylation (Figure 7d). Since light chain phosphorylation has been shown to regulate blebbing [32], [33], our results suggested that KSHV induced MLC II may be participating in the induction of blebbing during infection.

### c-Cbl is required for the recruitment of myosin IIA to the membrane blebs

During virus induced and apoptotic membrane blebbing, the signaling molecules associated with cytoskeletal function are recruited to the blebs [23], [26]. It has been demonstrated that the recruitment of functional myosin II heavy and light chain complexes drive the process of bleb retraction [26]; however, it is not clear how individual myosin II molecules are recruited to the blebs. It is possible that c-Cbl interaction with myosin IIA leads to recruitment of the complex to the blebs. Therefore, we infected control shRNA and c-Cbl shRNA cells with KSHV and tested the association of c-Cbl with myosin II in the blebs.

**Figure 8:**
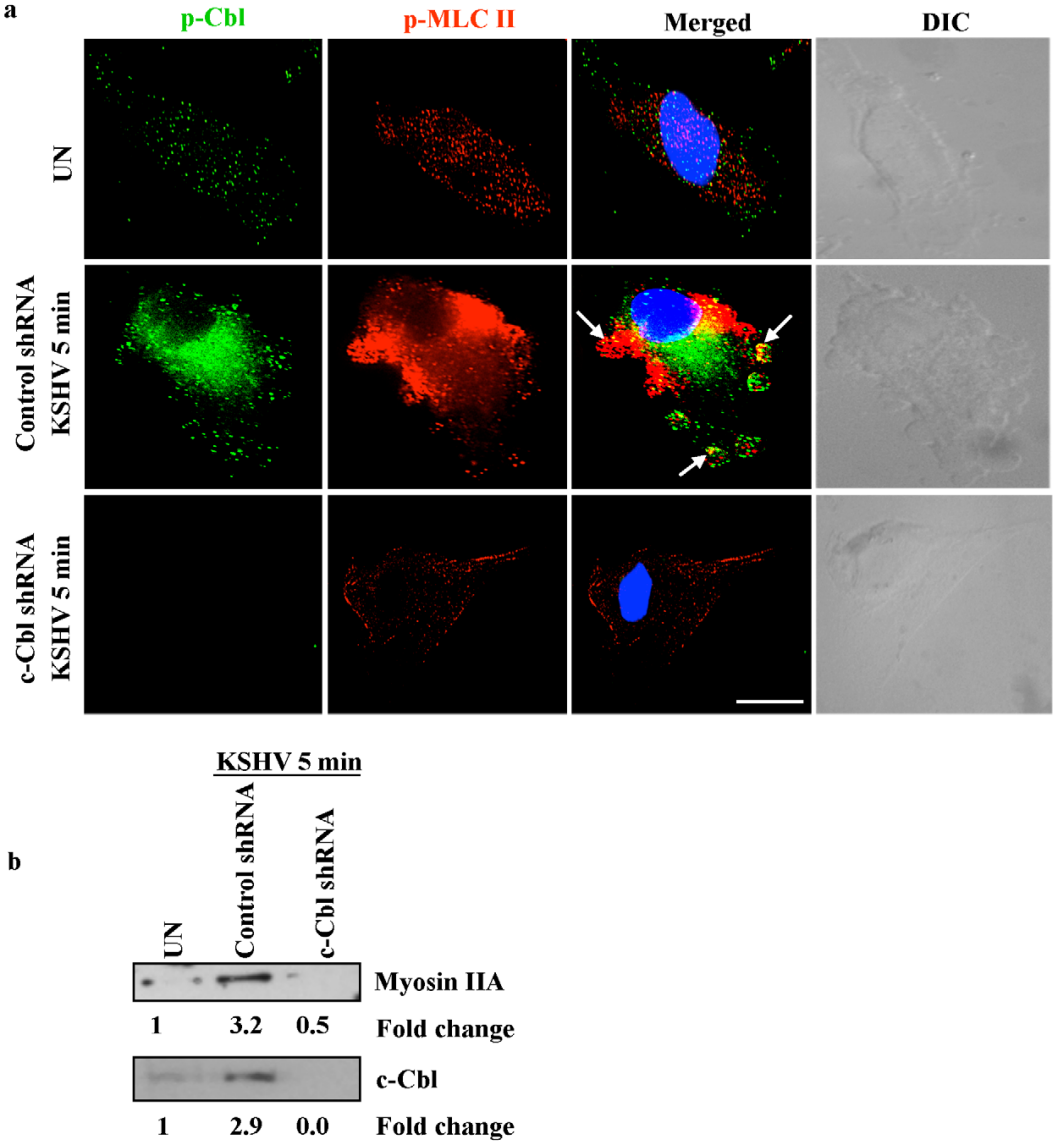
c-Cbl recruits myosin IIA to the membrane blebs. (a) Colocalization of c-Cbl and p-MLC II in KSHV induced blebs. Control and c-Cbl shRNA cells were infected with KSHV for 5 min. Uninfected and infected cells were then immunostained with anti-p-Cbl or anti-p-MLC II antibody. The rightmost panels show DIC images. In the merged panel, arrows indicate the colocalization of p-Cbl with p-MLC II. Scale bar: 10 µm. (b) Membrane association of myosin II in c-Cbl shRNA cells. Control shRNA and c-Cbl shRNA cells were infected with KSHV for 10 min. The membrane fractions were isolated from uninfected and infected samples, then Western blotted with myosin IIA to analyze the membrane localization of myosin IIA (top). The same membrane was stripped and reprobed with anti-c-Cbl to analyze the corresponding c-Cbl membrane localization (bottom).

Punctate staining of p-Cbl and p-MLC II was observed in the interior of blebs with a predominant colocalization between p-Cbl and p-MLC II in control shRNA infected cells (Figure 8a) suggesting that phosphorylated Cbl recruits individual myosin molecules to the blebs. In contrast, c-Cbl shRNA cells infected with KSHV did not show bleb formation and the recruitment and localization of myosin II to the bleb membrane (Figure 8a).

To further confirm the membrane localization of myosin IIA and c-Cbl to the blebs, membrane fractions from control shRNA infected cells and c-Cbl shRNA infected cells were isolated and analyzed by Western blotting. Compared to the uninfected cells, control shRNA cells infected with KSHV showed a 3.2 and 2.9-fold increase in membrane localization of myosin IIA and c-Cbl, respectively, whereas in c-Cbl shRNA-KSHV cells, membrane localization of myosin IIA and c-Cbl was almost completely absent (Figure 8b). This suggested that a decrease in membrane localization of myosin IIA in c-Cbl shRNA cells may be caused by a deficiency in the association of c-Cbl with myosin IIA.

### Myosin II associates with actin in KSHV induced blebs

**Figure 9:**
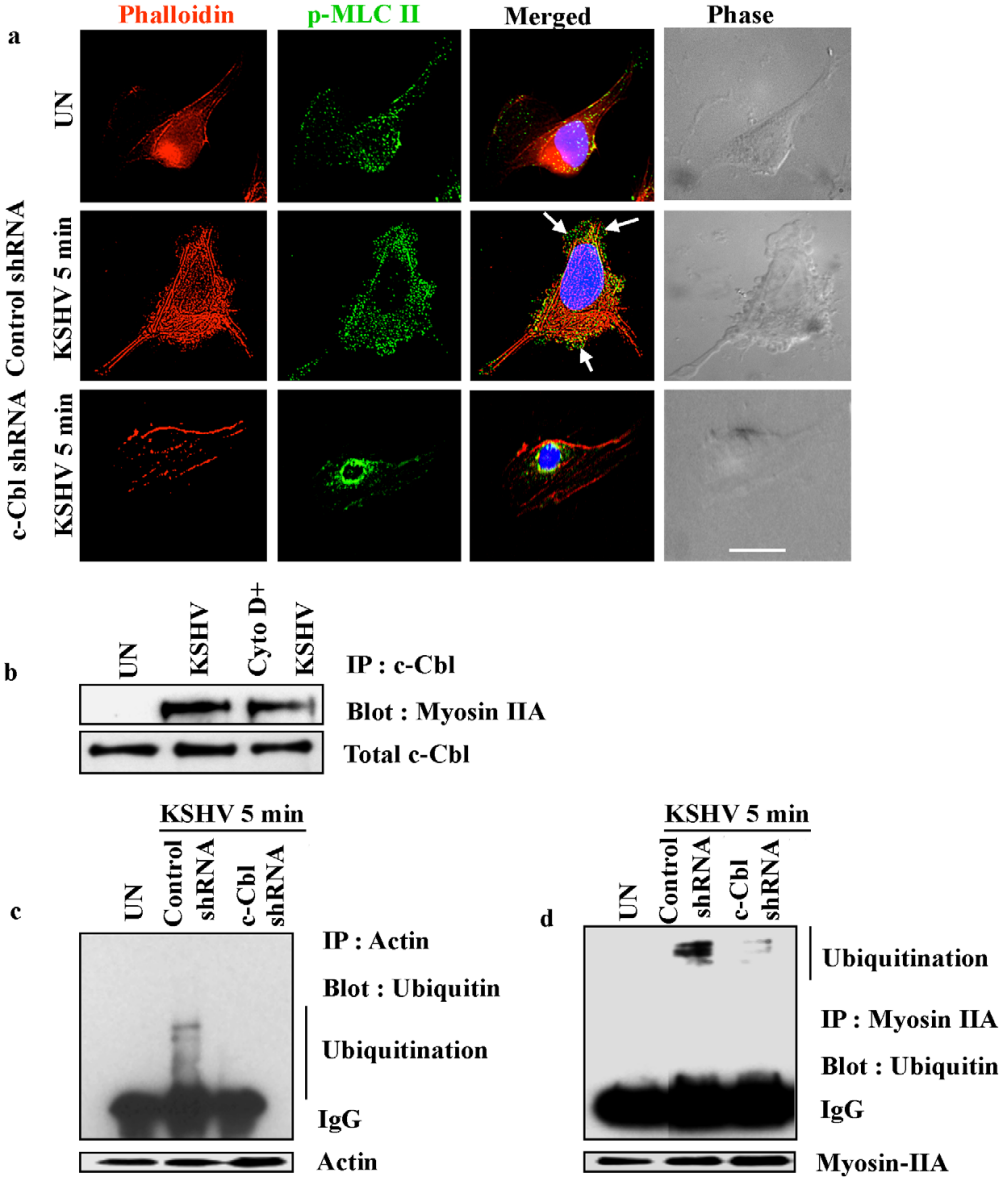
Myosin II associates with actin in KSHV induced blebs. (a) Colocalization of p-MLC II with actin in KSHV induced blebs. Control and c-Cbl shRNA transduced cells were infected with KSHV for 5 min and stained with rhodamine-phalloidin and anti-p-MLC II antibody. Arrows indicate the colocalization of p-MLC II and actin in the blebs. The rightmost column shows DIC images. Scale bar: 10 µm. (b) Actin inhibitor does not affect the interaction of myosin IIA with c-Cbl. HMVEC-d cells were pretreated with 2 µg ml^−1^ cytochalasin D for 1 h at 37°C and then infected with KSHV and washed. Cells were lysed and the cell lysates were immunoprecipitated with c-Cbl antibody and Western blotted with myosin IIA. (c) and (d) Ubiquitination of myosin and actin. Control shRNA and c-Cbl shRNA cells were serum starved overnight and infected with KSHV for 10 min, and lysed. Cell lysates were immunoprecipitated with anti-actin antibody (c) or anti-myosin antibody (d) and analyzed by Western blotting using anti-ubiquitin antibody. Membranes were reprobed with actin or myosin antibody. Arrow marks indicate multiple bands of ubiquitinated proteins. To show the multiple bands of ubiquitinated actin and myosin, actin (40 kDa) was resolved on a 12.5% gel (Figure 9c). Since myosin is a high molecular weight protein (220 kDa), for better resolution, 7.5% gel was used (Figure 9d).

Myosin II molecules recruited to the membrane blebs form contractile foci in association with actin under the bleb membrane which is critical for bleb retraction [26]. Therefore, we asked whether a similar kind of association occurs between p-MLC II and actin in control shRNA-KSHV and c-Cbl shRNA-KSHV cells. As has been previously reported [26], we observed the association of actin and p-MLC II in the membrane blebs in control shRNA KSHV infected cells (Figure 9a). Bleb formation and the association of actin with p-MLC II in the blebs were not seen in c-Cbl shRNA-KSHV cells (Figure 9a). The association of p-MLC II with actin, which is a known interacting partner of c-Cbl [10], coupled with the detection of an association with c-Cbl in the infected cells (Table S1) suggested that actin, myosin IIA and c-Cbl are part of a signaling complex which might be essential for the formation of blebs and bleb retraction.

To examine whether the interaction of c-Cbl with myosin IIA is actin dependent, we investigated the interaction of c-Cbl with myosin IIA in cells treated with actin inhibitor cytochalasin D. The interaction between c-Cbl and myosin IIA was then examined by coimmunoprecipitation and Western blot analysis. As shown in Figure 9b, cytochalasin D did not inhibit the interaction of myosin IIA with c-Cbl. The interaction of myosin IIA with c-Cbl in cytochalasin D treated cells suggested that actin is not essential for the initial association of myosin IIA with c-Cbl in KSHV infected cellular environment.

c*-*Cbl is an E3 ubiquitin ligase, which has been shown to be involved in poly or monoubiquitination of a number of proteins [34], [35], [36]. To further analyze the functional significance of the interaction between c-Cbl, myosin and actin, the role of c-Cbl in ubiquitination of actin and myosin was analyzed. Both myosin and actin ubiquitination were determined in control shRNA and c-Cbl shRNA cells infected with KSHV. We observed multiple bands of ubiquitinated myosin and actin probably indicating monoubiquitination on multiple sites and not polyubiquitination in the infected cells (Figure 9c and d). c-Cbl shRNA abolished the KSHV induced ubiquitination of actin and myosin suggesting that it was mediated by c-Cbl. This indicated that upon KSHV infection, c-Cbl binds to actin and myosin which in turn is responsible for ubiquitination.

## Discussion

Several viruses utilize macropinocytosis to gain access to target cells [24]. Macropinocytosis is strictly an actin driven process which includes the formation of membrane ruffles, lamellipodia and blebs [24]. Blebbing is a phenomenon which is mainly observed during cellular processes such as embryogenesis, cytokinesis and apoptotic cell death [25]. Bleb associated macropinocytosis provides a mechanism of virus entry into host target cells and has been shown to be an efficient tactic of the virus to enter the target cells by mimicking the apoptotic cells [23].

Macropinocytosis has been observed as a major route of entry of KSHV into HMVEC-d cells, the natural target cells of infection [3], [4]. During KSHV infection, the interaction of KSHV glycoproteins with integrins and other cellular receptors activate host cell signaling molecules FAK, Src, PI3-K, RhoA GTPase and are all recruited to the entry site [3]. The inhibition of any of these proteins significantly reduces the entry of virus suggesting that KSHV exploits preexisting host cell signaling machinery for a successful infection [3]. Our current study shows that c-Cbl is also required for efficient macropinocytic uptake, suggesting that the previously observed signaling molecules are linked via c-Cbl to perform their downstream functions. Since bleb associated macropinocytosis is an actomyosin dependent process, c-Cbl and its interaction with myosin playing a role in macropinocytosis further strengthens the possibility that c-Cbl is a critical molecule involved in linking the signaling molecules in the virus induced macropinocytic process.

Based on the strong evidences presented here, we propose that efficient bleb mediated macropinocytic uptake enables a productive infection of KSHV to occur in cells supporting a signaling cascade that contains the c-Cbl-myosin IIA complex, and that a defect in c-Cbl-myosin IIA association results in lowered macropinocytic uptake, entry and infection. The simultaneous decrease in macropinocytic uptake and blebbing by blebbistatin treatment and c-Cbl silencing with shRNA strongly suggests that bleb associated macropinocytosis is the predominant pathway of KSHV infection in HMVEC-d cells. The defect in macropinocytosis in c-Cbl shRNA cells could be due to a defect in linking myosin II molecules to the membrane associated events which is necessary for blebbing and bleb mediated macropinocytosis [24], [25], [26].

**Figure 10:**
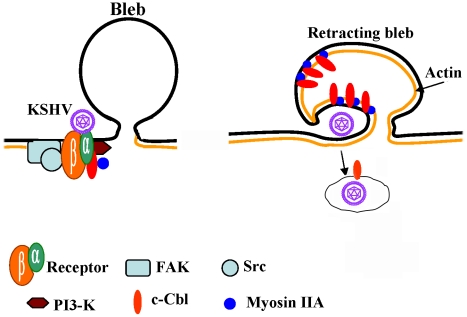
Model depicting the role of c-Cbl and myosin IIA interaction during blebbing and macropinocytosis of KSHV. KSHV has been previously shown to interact with heparin sulfate, α3β1, αVβ3 and αVβ5 integrins and xCT molecules leading to the phosphorylation of FAK and the downstream molecules such as Src, PI3-K and RhoGTPases [3]. KSHV infection induces the phosphorylation of c-Cbl and the phosphorylated c-Cbl forms a complex with p85-PI3K, thus facilitating the interaction of c-Cbl with downstream molecules. c-Cbl associates with myosin IIA and actin and is rapidly recruited to membrane blebs. This association also leads into c-Cbl mediated ubiquitination of actin and myosin. Myosin IIA interactions with actin may be accelerating the actomyosin contraction and bleb retraction which form macropinosomes along with the viral particles. Myosins probably provide the ATP-dependent force to generate the movement required for the process of bleb retraction. An increase in c-Cbl–myosin IIA association possibly affects c-Cbl-mediated blebbing, macropinocytosis and internalization of KSHV.

Although the molecular details of bleb associated macropinosome formation are yet to be uncovered, our study demonstrates that during KSHV infection, the interaction of c-Cbl with myosin IIA leads to bleb formation and the recruitment of myosin IIA into the blebs, where the myosin IIA molecules could be interacting with actin to accelerate actomyosin contraction and bleb retraction [26]. Retracting blebs form macropinosomes along with the viral particles close to the blebs (Figure 10) [24]. Myosins provide the ATP-dependent force to generate the movement required for the process of bleb retraction [26]. Myosin IIA is the major isoform of myosin II implicated in membrane associated functions such as the maintenance of cell shape and movement [37], [38]. The functional ending of ubiquitination is related to the type of ubiquitin chains added to the substrate protein [39]. Monoubiquitination promotes internalization of cell surface receptors and subsequent lysosomal degradation [40], whereas polyubiquitinated proteins are targeted for proteasomal degradation [41]. Whether the complex events associated with the monoubiquitination of actin and myosin by c-Cbl could be related to an increase in the activity of actomyosin contraction or directing subcellular compartmentalization during KSHV infection and macropinocytosis remains to be studied in detail. Further studies are required to understand the molecular aspects of the interaction between c-Cbl and myosin IIA and their role in subsequent stages of bleb mediated macropinocytosis. Further studies are also required to validate the specificity and the functional significance of other identified c-Cbl interacting proteins, their association with c-Cbl during infection and whether c-Cbl induces the ubiquitination of cell surface molecules recognized by KSHV.

In conclusion, our results provide for the first time clear evidence demonstrating that c-Cbl, and the interaction between c-Cbl and myosin IIA, is critical for triggering bleb mediated macropinocytic events during KSHV entry into target cells (Figure 10). This study also provides the first evidence that c-Cbl and a c-Cbl mediated signaling pathway as well as ubiquitination play roles in viral infection and that c-Cbl function as an adaptor protein for PI3-K and other KSHV induced signaling events. This also identifies c-Cbl as a potential target to intervene in KSHV infection.

## Supporting Information

Figure S1 (a) shRNA transduction. HMVEC-d cells transduced with c-Cbl shRNA lentivirus or with non-targeting control shRNA lentivirus were selected using puromycin hydrochloride. Western blotting with anti c-Cbl antibody showed 90% knockdown of endogenous c-Cbl expression in c-Cbl shRNA transduced cells. (b and c) Histograms depict KSHV ORF73 (b) and ORF50 (c) gene RNA copy numbers in untransduced, control shRNA and c-Cbl shRNA transduced cells. Each reaction was done in duplicate, and each bar represents the mean ± SD of the results of three independent experiments. (d) Histogram shows KSHV internalized viral ORF73 DNA copy numbers in untransduced, control shRNA and c-Cbl shRNA transduced cells. Each reaction was done in duplicate, and each bar represents the mean ± SD of three experiments.
(0.29 MB TIF)

Figure S2 Actin reorganization and blebbing in KSHV infected cells. HMVEC-d cells were either left uninfected or infected with KSHV for 2 or 5 min, stained with rhodamine-phalloidin for filamentous actin and anti-gB (viral glycoprotein) antibody for the detection of KSHV. Arrows indicate viral particles stained with gB; arrow heads indicate blebs formed (devoid of an actin cortex) during KSHV infection; blocked arrows indicate a retracting bleb (with thick actin cortex) with viral particles. Scale bar: 10 µM. The boxed areas are enlarged in the rightmost column.
(1.74 MB TIF)

Figure S3 Uptake and colocalization of dextran with KSHV. (a) Representative immunofluorescence image for figure 4b showing dextran uptake in uninfected and infected control and c-Cbl shRNA cells. Dextran positive cells are merged wth DAPI in the merged panel. (b) Immunofluorescence image of a microscopic field of view showing dextran uptake and colocalization with KSHV in control and c-Cbl shRNA cells. Right- most panels show enlarged single cell from each panel. Dashed lines represent the cell periphery. Arrows indicate uptake and colocalization of dextran and KSHV in control cells; arrowheads indicate viral particles that remained at the cell periphery in c-Cbl shRNA cells; blocked arrows indicate non colocalization of transferrin and KSHV in control cells.
(1.90 MB TIF)

Figure S4 Schematic illustration of HA tagged Cbl wild-type, Cbl-TKB mutant (which lacks a functional TKB domain), Cbl-RING mutant (which lacks ubiquitin ligase activity of the RING domain), and two truncation mutants Cbl-Δ357 and Cbl-Δ421 (which lacks the C-terminal proline rich domain as well as the UBA and LZ domain).
(0.12 MB TIF)

Table S1 Mass spectrometry analysis of c-Cbl interacting proteins in the infected samples. c-Cbl interacting proteins identified in mass spectrometry analysis. Serum starved HMVEC-d cells were left uninfected or infected with KSHV for 1, 5, 10 min, and the cell lysates were immunoprecipitated with anti-c-Cbl antibody. Immunoprecipitated proteins were separated by SDS-PAGE gel and the gel slices were analyzed by mass spectrometry. The table shows a list of proteins identified by mass spectrometry. The score (%) and coverage (%) obtained for each protein are also indicated.
(0.05 MB RTF)
