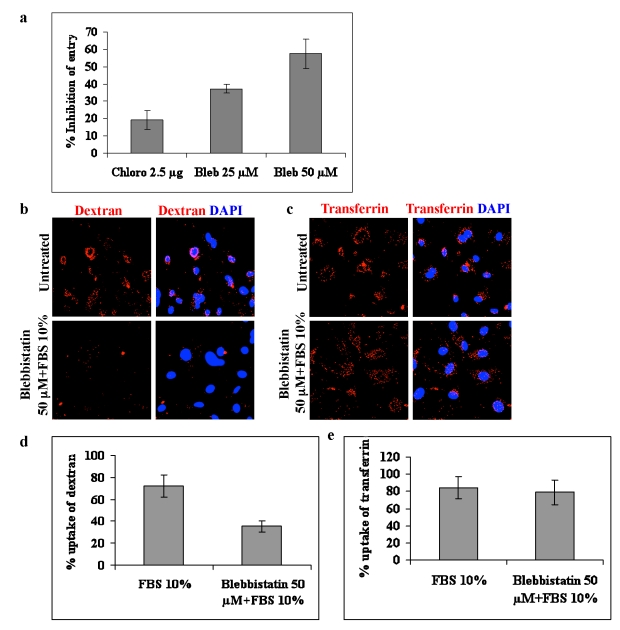# Correction: Interaction of c-Cbl with Myosin IIA Regulates Bleb Associated Macropinocytosis of Kaposi's Sarcoma-Associated Herpesvirus

**DOI:** 10.1371/annotation/79109603-41dd-40b8-a9ec-8df2c7fa42eb

**Published:** 2011-01-28

**Authors:** Mohanan Valiya Veettil, Sathish Sadagopan, Nagaraj Kerur, Sayan Chakraborty, Bala Chandran

Section E of Figure 6 is incorrect. Please see the corrected Figure 6 here: 

**Figure:**