# Supplementary figures and images for: Interaction of c-Cbl with Myosin IIA Regulates Bleb Associated Macropinocytosis of Kaposi's Sarcoma-Associated Herpesvirus
Source: PLoS Pathog. 2010 Dec 23;6(12):e1001238. doi: 10.1371/journal.ppat.1001238 (PMC3009604; doi:10.1371/journal.ppat.1001238)

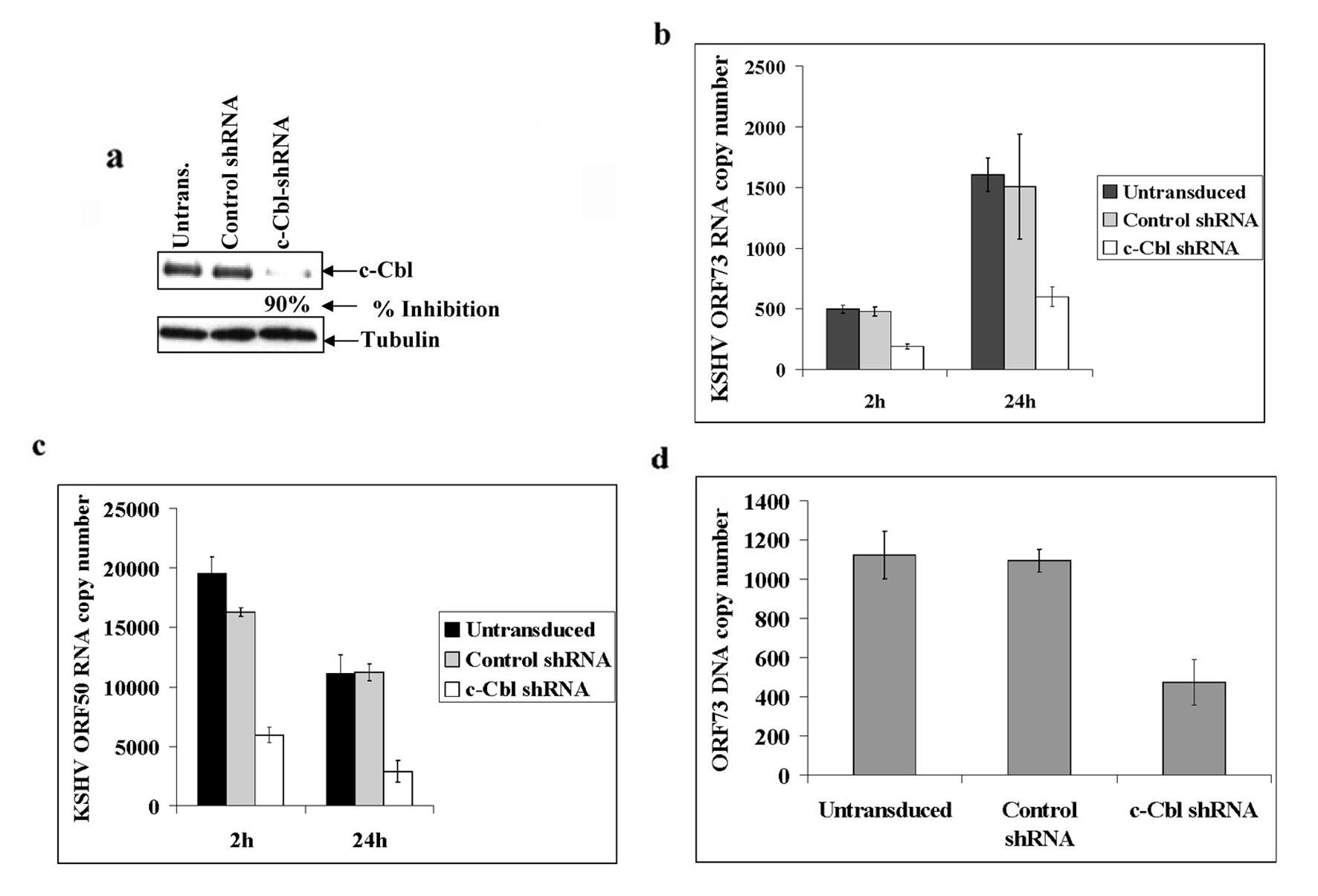

Supplement: Figure S1 — (a) shRNA transduction. HMVEC-d cells transduced with c-Cbl shRNA lentivirus or with non-targeting control shRNA lentivirus were selected using puromycin hydrochloride. Western blotting with anti c-Cbl antibody showed 90% knockdown of endogenous c-Cbl expression in c-Cbl shRNA transduced cells. (b and c) Histograms depict KSHV ORF73 (b) and ORF50 (c) gene RNA copy numbers in untransduced, control shRNA and c-Cbl shRNA transduced cells. Each reaction was done in duplicate, and each bar represents the mean ± SD of the results of three independent experiments. (d) Histogram shows KSHV internalized viral ORF73 DNA copy numbers in untransduced, control shRNA and c-Cbl shRNA transduced cells. Each reaction was done in duplicate, and each bar represents the mean ± SD of three experiments. (0.29 MB TIF) [file ppat.1001238.s001.tif]

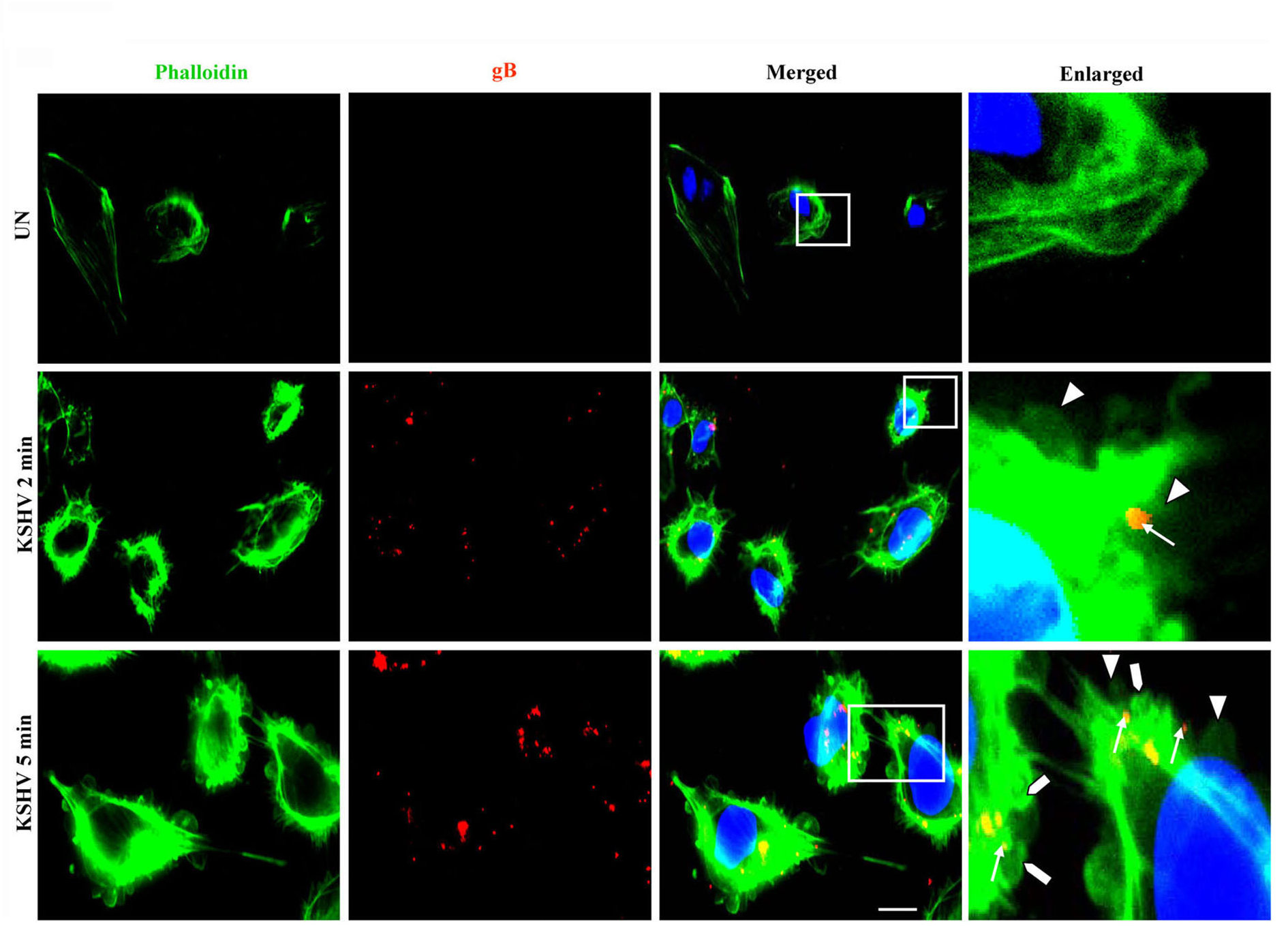

Supplement: Figure S2 — Actin reorganization and blebbing in KSHV infected cells. HMVEC-d cells were either left uninfected or infected with KSHV for 2 or 5 min, stained with rhodamine-phalloidin for filamentous actin and anti-gB (viral glycoprotein) antibody for the detection of KSHV. Arrows indicate viral particles stained with gB; arrow heads indicate blebs formed (devoid of an actin cortex) during KSHV infection; blocked arrows indicate a retracting bleb (with thick actin cortex) with viral particles. Scale bar: 10 µM. The boxed areas are enlarged in the rightmost column. (1.74 MB TIF) [file ppat.1001238.s002.tif]

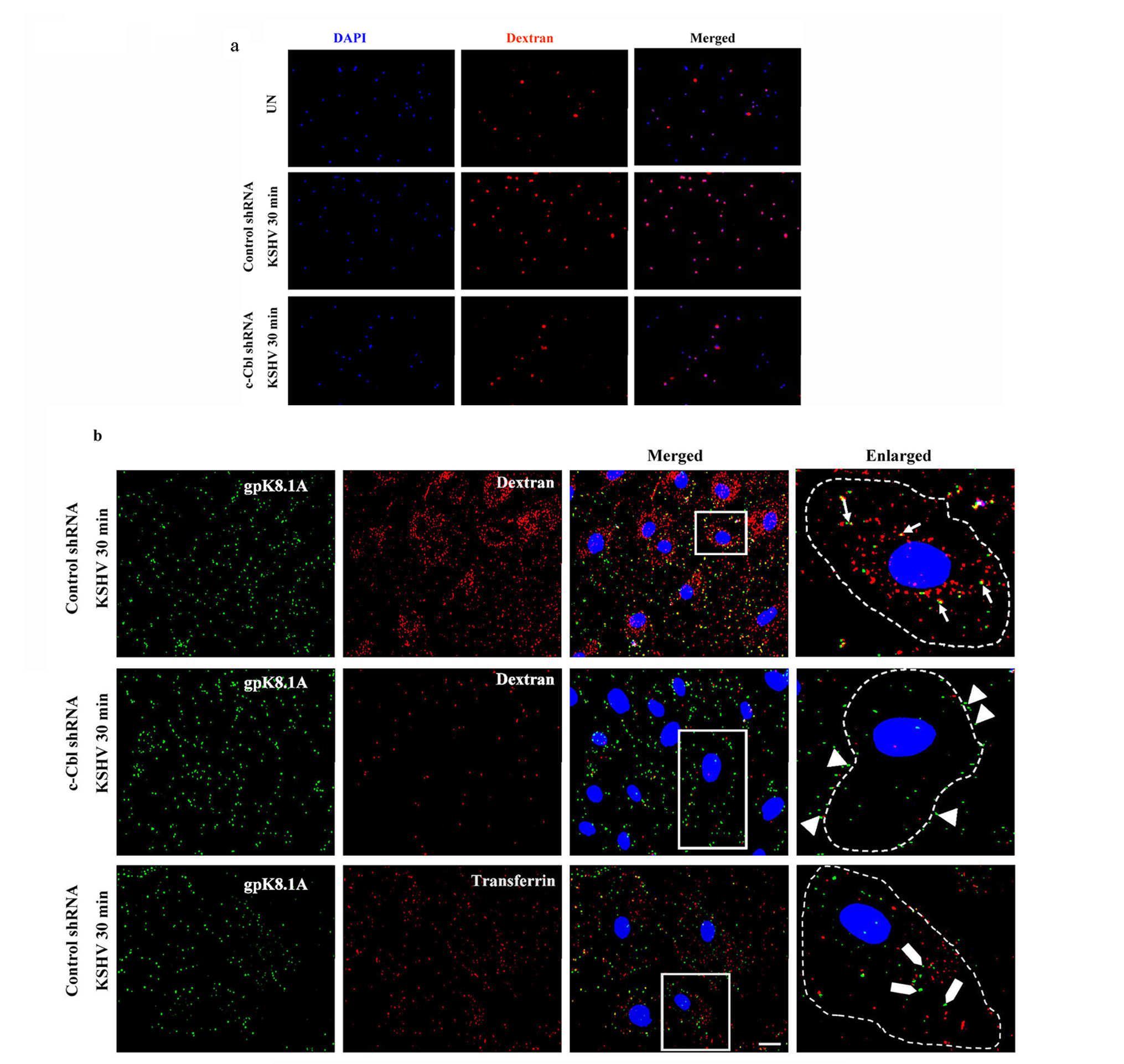

Supplement: Figure S3 — Uptake and colocalization of dextran with KSHV. (a) Representative immunofluorescence image for figure 4b showing dextran uptake in uninfected and infected control and c-Cbl shRNA cells. Dextran positive cells are merged wth DAPI in the merged panel. (b) Immunofluorescence image of a microscopic field of view showing dextran uptake and colocalization with KSHV in control and c-Cbl shRNA cells. Right- most panels show enlarged single cell from each panel. Dashed lines represent the cell periphery. Arrows indicate uptake and colocalization of dextran and KSHV in control cells; arrowheads indicate viral particles that remained at the cell periphery in c-Cbl shRNA cells; blocked arrows indicate non colocalization of transferrin and KSHV in control cells. (1.90 MB TIF) [file ppat.1001238.s003.tif]

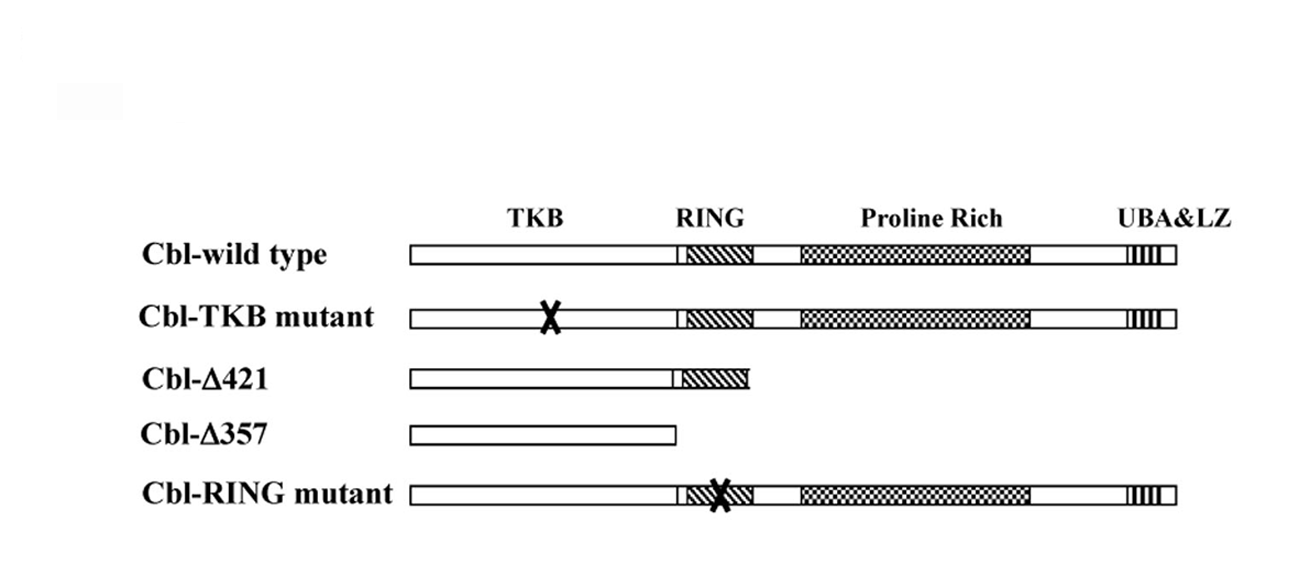

Supplement: Figure S4 — Schematic illustration of HA tagged Cbl wild-type, Cbl-TKB mutant (which lacks a functional TKB domain), Cbl-RING mutant (which lacks ubiquitin ligase activity of the RING domain), and two truncation mutants Cbl-Δ357 and Cbl-Δ421 (which lacks the C-terminal proline rich domain as well as the UBA and LZ domain). (0.12 MB TIF) [file ppat.1001238.s004.tif]
