# Supplementary material for: Interaction of c-Cbl with Myosin IIA Regulates Bleb Associated Macropinocytosis of Kaposi's Sarcoma-Associated Herpesvirus
Source: PLoS Pathog. 2010 Dec 23;6(12):e1001238. doi: 10.1371/journal.ppat.1001238 (PMC3009604; doi:10.1371/journal.ppat.1001238)
Supplement: Table S1 — Mass spectrometry analysis of c-Cbl interacting proteins in the infected samples. c-Cbl interacting proteins identified in mass spectrometry analysis. Serum starved HMVEC-d cells were left uninfected or infected with KSHV for 1, 5, 10 min, and the cell lysates were immunoprecipitated with anti-c-Cbl antibody. Immunoprecipitated proteins were separated by SDS-PAGE gel and the gel slices were analyzed by mass spectrometry. The table shows a list of proteins identified by mass spectrometry. The score (%) and coverage (%) obtained for each protein are also indicated. (0.05 MB RTF) [file ppat.1001238.s005.rtf]

Table S1: Mass spectrometry analysis of c-Cbl interacting proteins in the infected samples

Name of the protein	Score (%)	Coverage (%)	
Myosin heavy chain IIA	99	12.7	
Beta actin	98.6	9.5	
Alpha actin	98.2	8	
ACTB protein (also called actin)	98.6	9.7	
ACTG2 (also called actin)	98.2	7.9	
Vimentin	98.3	6.2	
HSP70	98.7	9.7	
BiP protein 	98.1	8.5	
RhoGEF	55.3	5.6	
Solute carrier anion exchanger	46.1	4.0	
Cytokeratin	55	4.4	

Table S1 legend: c-Cbl interacting proteins identified in mass spectrometry analysis. Serum starved HMVEC-d cells were left uninfected or infected with KSHV for 1, 5, 10 min, and the cell lysates were immunoprecipitated with anti-c-Cbl antibody. Immunoprecipitated proteins were separated by SDS-PAGE gel and the gel slices were analyzed by mass spectrometry. The table shows a list of proteins identified by mass spectrometry. The score (%) and coverage (%) obtained for each protein are also indicated. 
